# Elucidating the antimicrobial and anticarcinogenic potential of methanolic and water extracts of edible *Tragopogon coelesyriacus* Boiss.

**DOI:** 10.1002/fsn3.4341

**Published:** 2024-07-17

**Authors:** Tuba Unver, Ugur Uzuner, Selcen Celik‐Uzuner, Ismet Gurhan, Nur Sena Sivri, Zeynep Ozdemir

**Affiliations:** ^1^ Department of Pharmaceutical Microbiology, Faculty of Pharmacy Inonu University Malatya Turkey; ^2^ Department of Molecular Biology and Genetics, Faculty of Science Karadeniz Technical University Trabzon Turkey; ^3^ Department of Pharmaceutical Botany, Faculty of Pharmacy Inonu University Malatya Turkey; ^4^ Department of Pharmaceutical Chemistry, Faculty of Pharmacy Inonu University Malatya Turkey

**Keywords:** anticancer activity, antimicrobial activity, flavonoid glycosides, molecular docking, pharmacotherapeutic plant, *Tragopogon coelesyriacus*

## Abstract

*Tragopogon coelesyriacus* is a pharmacotherapeutic herbaceous plant belonging to the Asteraceae family and consumed as a vegetable. Here, the methanolic and water extracts of *T. coelesyriacus* were obtained from its aboveground parts (stem, leaves, and flowers), and the phytochemical potentials were investigated by LC‐HRMS (liquid chromatography–high resolution mass spectrometry) analysis for the first time. The antibacterial, antifungal, and anticarcinogenic activities of *T. coelesyriacus* extracts were investigated using experimental and in silico methods. *T. coelesyriacus* methanol extract revealed remarkable inhibitory activity against *Staphylococcus aureus*, *Pseudomonas aeruginosa*, and *Klebsiella pneumonia* (MICs = 0.83, 1.67, and 1.67 mg/mL, respectively) compared to *Escherichia coli* and *Enterobacter aerogenes* (MIC = 53.3 mg/mL). Inhibitory effects of *T. coelesyriacus* methanolic extracts were also observed in all *Candida* species tested, with the highest inhibition on *Candida krusei* (MIC = 0.83 mg/mL), whereas no inhibitory effect was identified from the water extract. Additionally, both *T. coelesyriacus* methanolic (IC50 = 86 μg/mL) and water (IC50 = 92 μg/mL) extracts revealed significant selective anticarcinogenic effects on AR42J pancreatic cancer cells. HeLa and MDA‐MB‐231 cells were, however, more resilient to methanol and water extract, respectively. In silico analyses further elucidated the noteworthy antibacterial potential of keracyanin chloride on *S. aureus* MurB enzyme and the remarkable inhibitory potential of naringin on FYN kinase specific for pancreatic cancer (AR42J) development. In conclusion, *T. coelesyriacus* phytochemicals with antibacterial, antifungal, and anticancer properties were revealed for the first time, and molecular docking studies on potential targets confirmed good agreement with experimental findings. Therefore, the current studies on *T. coelesyriacus* provide the basis for investigating new pharmaceutical potentials of other *Tragopogon* members.

## Graphical abstract

*Tragopogon coelesyriacus* methanol extract has a significant antimicrobial effect, predominantly against *S. aureus* and *C. krusei*. The plant extracts have a cytotoxic effect on AR42J pancreatic cancer cells, while HeLa cervical and MDA‐MB‐231 breast cancer cells were more resistant. Keracyanin chloride has an inhibitory effect on *S. aureus* MurB protein and an inhibitory potential of the Naringin molecule on FYN kinase protein. Consequently, *T. coelesyriacus* can be used as a potent pharmacological and therapeutic‐protective agent.

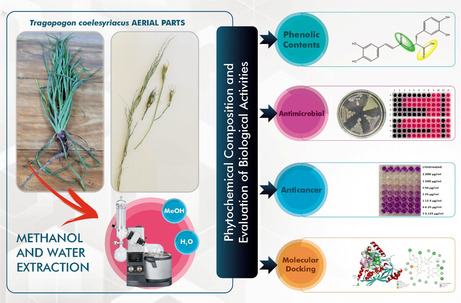

## 1 INTRODUCTION

The use of phytotherapeutic plants in health care is as old as human history and dates back thousands of years (Petrovska, 2012). In the last century, considering the severe side effects of chemical antibiotics and anticancer drugs, the popularity of these plants in traditional medicine has increased. The negativities of infectious diseases caused by pathogenic microorganisms and antibiotic resistance to treatment constitute serious problems worldwide (Aslam et al., 2018; Dadgostar, 2019; MacPherson et al., 2009; Simonsen, 2018). Therefore, research on medicinal plants has intensified to discover bioactive plant extracts or compounds against microorganisms. In addition, cancer has an increased mortality rate and is the second leading cause of death worldwide after cardiovascular diseases (GBD, 2015; Siegel et al., 2015). In cancer treatment, resistance to cancer chemotherapy and severe side effects of chemotherapeutic drugs have led people to herbal treatment methods (Alfarouk et al., 2015). Anticancer studies with herbal substances have proven that thousands of plant species have anticancer effects worldwide (Abdalla & Zidorn, 2020; Graham et al., 2000; Kumar et al., 2022; Salaroli et al., 2022). In recent studies with species belonging to the *Tragopogon* genus, it has been stated that this plant may have phytotherapeutic activity (Abdalla & Zidorn, 2020; Bahadır Acıkara et al., 2013; Sarac, 2015; Tabaraki et al., 2013).

*Tragopogon* (Asteraceae) is represented by 150 species, native to the semiarid region and growing mainly in Europe and Asia (Bell et al., 2012). In Turkey, it is known as a manger or goatee and is consumed as a vegetable by the people of Eastern and Southeastern Anatolia (Baytop, 2000). The members of the *Tragopogon* species are distributed with 26 taxa belonging to approximately 22 species in Turkey (Gültepe et al., 2021; Güner et al., 2012). The leaves of the *Tragopogon coelesyriacus* are grass‐like, the stems have few branches, and they secrete a milky sap if the stems or leaves are broken (Richardson, 1976). In Turkish folk medicine, it has been found that some *Tragopogon* species have anthelminthic activities and are used to treat abdominal pain. In addition, the leaves and stem of *T. coelesyriacus* are used in intestinal disorders (Altundag & Ozturk, 2011; Ugur et al., 2010). A study revealed a comparative collection of ancient *materia medica* (a pharmacopeia of drugs written in the first century AD), which includes hundreds of medicinal plants mentioned in the literature. *T. coelesyriacus* is also included among this wide variety of plants used in traditional medical sciences. It is known that almost all of the natural medicinal substances used in the middle ages have been used for popular healing purposes in the last 200 years. The most crucial evidence is that folk healers in the Middle East and the Far East still use these methods (Lev, 2002).

In studies with extracts of *Tragopogon* species, although it has been observed that this plant has antitumor, anti‐inflammatory, antimicrobial, wound healing, enzyme inhibitor, antihyperlipidemic, and hepatoprotective effects, there are not enough studies on the toxicity assessment and chemical composition of the extracts (Abdalla & Zidorn, 2020). These effects have been associated with compounds in the plant crude extracts. Flavonoids, terpenoids, and several natural products associated with various classes have been determined in numerous taxa of the genus *Tragopogon* (Lusa et al., 2016; Miyase et al., 1992). Phenolic compounds such as chlorogenic acid, quinic acid, gallic acid, vitexin, ferulic acid, rosmarinic acid, and luteolin were detected in the extracts of *Tragopogon* species (Bayrami et al., 2018; Farzaei, Khanavi, et al., 2014; Granica et al., 2015; Lusa et al., 2016; Sareedenchai et al., 2009; Smolarz & Krzaczek, 1988; Uysal et al., 2019). The plant's antimicrobial and antioxidant properties are related to its phenolic compounds (Martínez‐González et al., 2017). Phenolic compounds show antimicrobial properties by damaging bacterial cell membranes and DNA during replication and inactivating bacterial enzymes (Kim et al., 2013). Moreover, phenolic compounds reduce the redox potential of the environment due to the antioxidant activities of hydroxyl groups and prevent the growth of many aerobic microorganisms (Bayram & Topuz, 2023). For this purpose, medicinal plants can be used to produce natural compounds with critical therapeutic properties (Gündüz et al., 2023).

In the literature, various antimicrobial and antitumor studies have been conducted on different species belonging to the *Tragopogon* genus. In the present study, the antitumorigenic potential of *T. coelesyriacus* extracts was also examined on three epithelial cancerous cells (AR42J, pancreatic cancer; HeLa, cervical cancer, and MDA‐MB‐231 breast cancer). In these studies, the inhibition values of different species of *Tragopogon* on various microorganisms varied, and anticancer activities were evaluated on different cell lines using different parts and extracts of plants (Kucekova et al., 2013; Moromete et al., 2016; Tenkerian et al., 2015; Uysal et al., 2019; Wegiera et al., 2012). However, this is the first study to demonstrate the antimicrobial and anticancer properties of *T. coelesyriacus*. Therefore, the present study aimed to reveal the antimicrobial and anticancer activity of water and methanol extracts of *T. coelesyriacus*, together with molecular docking assays. To elucidate the antibacterial and anticancer effects of *T. coelesyriacus* natural compounds, two‐way molecular docking studies were carried out. In silico molecular docking studies on *S. aureus* UDP‐*N*‐acetylenolpyruvylglucosamine reductase (MurB; PDB ID: 1HSK) were performed to investigate antibacterial activity potentials of *T. coelesyriacus* natural compounds. These in vitro and in silico results suggest that *T. coelesyriacus* can be used as a potent herbal pharmacological agent in medicine, pharmacy, food industry, and even cosmetics.

## 2 MATERIALS AND METHODS

### 2.1 Collection of *T. coelesyriacus* and preparation of extracts

*T. coelesyriacus* (previously known as *Tragopogon porrifolius* subsp. *longirostris* (Sch. Bip.) Greuter, *Tragopogon longirostris* Sch. Bip. var. *longirostris*) aerial parts (stem, leaf, and flower) were collected from Puturge‐Taslıpara district of Malatya province (Turkey) (Coordinates: 38°11′46.48″ N‐ 38°52′19.59″ E) in April and May 2022 and brought to Inonu University, Faculty of Pharmacy, Pharmaceutical Botany laboratory, and was identified in this laboratory (Matthews et al., 1975). The plant aerial parts were dried and then crushed with a grinder before extraction. Two different solvents (water and methanol) were used in the extraction. Ten grams of the aerial parts of the plants was weighed, and 100 mL of water or methanol was added to them and left to maceration at room temperature. This process was repeated three times by removing the filtrate and adding solvent. The collected filtrates were combined and evaporated in a rotary evaporator (Heidolph Laborota 4000, Germany) until the solvents were removed (Radulović et al., 2007). The remaining extracts were stored in a deep freezer at −18°C until used in studies.

### 2.2 LC‐HRMS (liquid chromatography–high‐resolution mass spectrometry) analysis

Liquid chromatography–high‐resolution mass spectrometry (LC‐HRMS) studies were carried out using an electrospray ionization (ESI) source and a reversed phase C18 column on an Agilent 6460 Triple Quad LC–MS/MS with a 1290 Infinity UPLC system (Abreu et al., 2023; Renda et al., 2023). According to the literature method, the elution gradient was formed up of two mobile phases: water and 0.1% formic acid, and acetonitrile and 0.1% formic acid. The column temperature remained stable at 30°C. The gradient elution was applied to mobile phase B at concentrations ranging from 5 to 5‐20‐90‐90‐5‐5 after 0‐4‐7‐14‐15‐15.1‐20 min, with a flow rate of 0.4 mL/min (Can et al., 2024). The compounds were determined and identified by comparing the retention durations of reference compounds with HRMS data from the Eastern Anatolia High Technology Application and Research Center. The mobile phase (1 mL; A: B; 50:50; v/v) was dissolved with 1 mg of dry water and methanol extract. Before being injected into the LC in a volume of 5 μL, the solution was filtered through a 0.45‐m filter.

### 2.3 Antibacterial and antifungal activities

#### 2.3.1 Strains

The following nine microorganisms, consisting of five bacteria (two Gram‐positive and three Gram‐negative) and four yeast, were studied: *Staphylococcus aureus* (ATCC 12600), *Enterobacter aerogenes* (ATCC 51697), *Pseudomonas aeruginosa* (ATCC 10145), *Escherichia coli* (C2987), *Klebsiella pneumoniae* (ATCC 13883), *Candida albicans* (ATCC 14053), *Candida tropicalis* (ATCC 13803), *Candida parapsilosis* (ATCC 22019), and *Candida krusei* (ATCC 14243). *E. coli* was purchased from New England Biolabs (NEB, MA, US). Other strains were purchased from the American Type Culture Collection (ATCC, VA, US).

#### 2.3.2 Media

Mueller–Hinton broth (MHB), Mueller–Hinton agar (MHA), Sabouraud dextrose agar (SDA), and Sabouraud dextrose broth (SDB) were used for the antimicrobial activity of water and methanol extracts of *T. coelesyriacus*. While MHA and MHB were used for bacterial strains, SDA and SDB were used for yeast strains.

#### 2.3.3 Agar dilution assay

**FIGURE 1:**
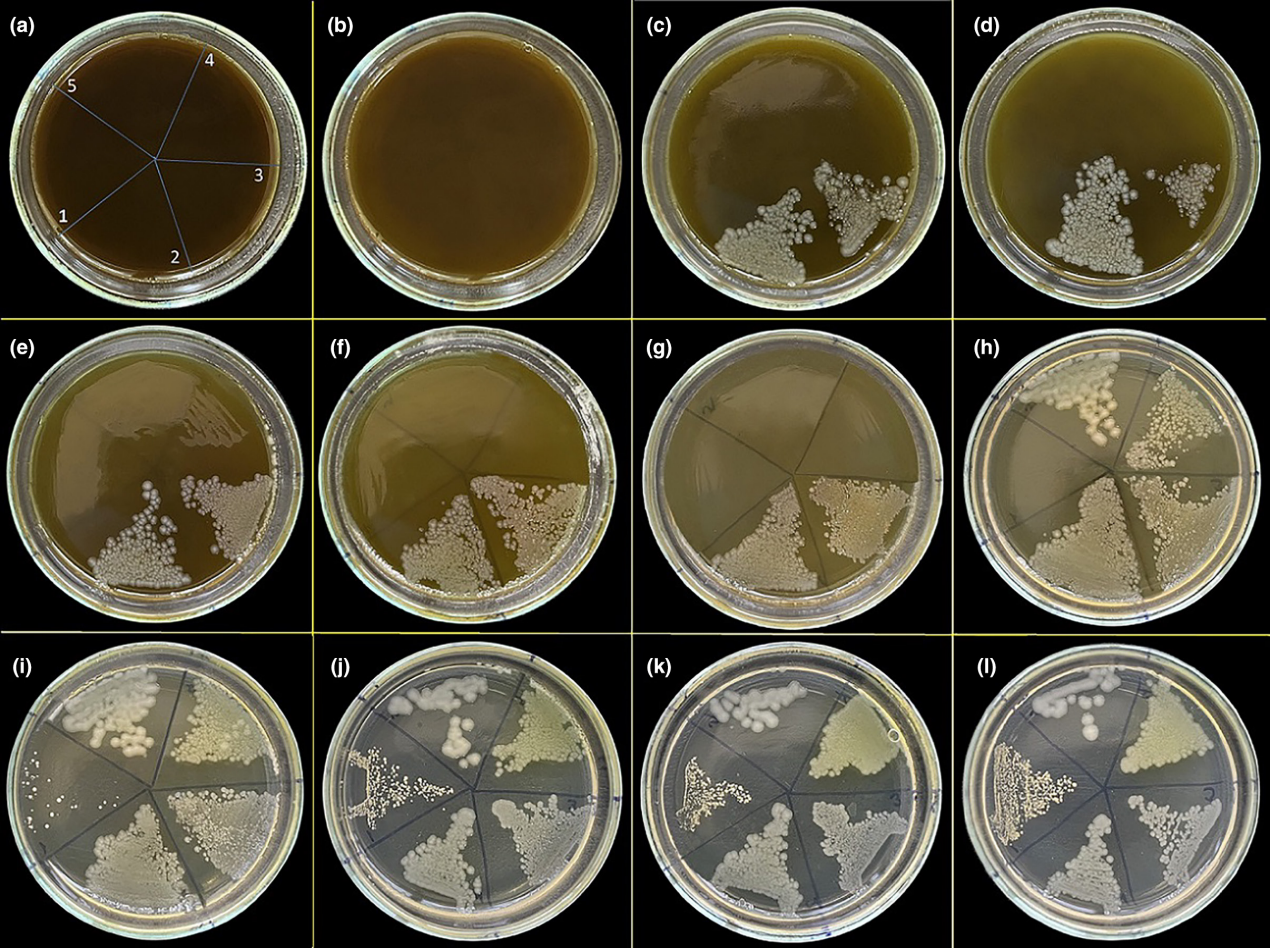
Petri dish pictures of the antibacterial activity assay of the *T. coelesyriacus* methanol extract with various concentrations against five bacterial strains: (1) *S. aureus*, (2) *E. aerogenes*, (3) *E. coli*, (4) *P. aeruginosa*, and (5) *K. pneumoniae*. (a) 106.6 (b) 53.3, (c) 26.6, (d) 13.3, (e) 6.6, (f) 3.3, (g) 1.67, (h) 0.83, (i) 0.42, (j) 0.21, (k) 0.1 mg/mL methanol extract of *T. coelesyriacus*, (l) Control.

The agar dilution assay was carried out to analyze the antimicrobial properties of *T. coelesyriacus* against nine different microorganisms, namely, *S. aureus*, *E. aerogenes*, *E. coli*, *P. aeruginosa*, *K. pneumoniae*, *C. tropicalis*, *C. albicans*, *C. krusei*, and *C. parapsilosis*. For two‐fold agar dilution method, 640, 320, 160, 80, 40, 20, 10, 5, 2.5, 1.25, 0.6 g (w/v) methanol and water *extracts of T. coelesyriacus* were added to 6‐mL agar plates separately to obtain the concentration of 106.6, 53.3, 26.6, 13.3, 6.6, 3.3, 1.67, 0.83, 0.42, 0.21, and 0.1 (mg/mL), respectively. While MHA was used for bacterial strains, SDA was used for yeast strains. Pure MHA and SDA were prepared separately without the extracts for control plates. Direct colony suspensions were prepared in distilled water for each species, and their turbidity was adjusted to 0.5 McFarland (1 × 10^8^ cfu/mL for bacterial strains, 1 × 10^6^ cfu/mL for yeast strains). Afterward, MHA plates were separated into five distinct regions: *S. aureus* to the first region, *E. aerogenes* to the second region, *E. coli* to the third region, *P. aeruginosa* to the fourth region, and *K. pneumoniae* to the fifth region. Furthermore, SDA plates were divided into four regions: *C. albicans* in the first region, *C. tropicalis* in the second region, *C. krusei* in the third region, and *C. parapsilosis* in the fourth region, as applied before (Unver, 2024a, 2024b; Unver et al., 2023). One microliter of each microorganism from the standard inoculum was inoculated onto the agar plates in each region, to which different *T. coelesyriacus* extract concentrations were added. The last plate, the L plate, shows the viability of microorganisms and contains a pure agar medium. In an incubator, inoculated media were incubated at 36°C overnight. Thereafter, the growth of each microorganism strain on the plates was evaluated, and the minimum inhibitory concentrations (MIC) were determined for each strain (Figure 1). The experiment was performed in triplicate.

#### 2.3.4 Broth microdilution method

In the broth two‐fold microdilution assay, the antibacterial and antifungal activity tests of *T. coelesyriacus* were repeated against nine different microorganisms, including *S. aureus*, *E. aerogenes*, *E. coli*, *K. pneumoniae*, *P. aeruginosa*, *C. albicans*, *C. tropicalis*, *C. krusei*, and *C. parapsilosis*, using a 96‐well microplate, as applied before (Unver et al., 2023, 2024). The amounts of 21.33, 10.66, 5.33, 2.66, 1.33, 0.66, 0.33, 0.16, 0.08, and 0.04 mg of methanol and water extracts of *T. coelesyriacus* were added to 200‐μL MHB for bacterial strains, and SDB for yeast strains in each well. For control groups, pure MHB and SDB without extracts were placed in one of the control wells as negative controls, and microorganisms used with MHB and SDB were placed in the other well as positive controls. Each microorganism's direct colony suspension was prepared in distilled water, and their turbidity was adjusted to standard inoculum 0.5 McFarland. Afterward, 1 μL of *S. aureus*, *E. aerogenes*, *E. coli*, *P. aeruginosa*, *K. pneumoniae*, *C. albicans*, *C. tropicalis*, *C. krusei*, and *C. parapsilosis* were inoculated into the A, B, C, D, E, F, G, H, and I row of the microplate, respectively. The microplate with different extracts of *T. coelesyriacus* concentrations was incubated at 36°C for 24 h, and subsequently, 15 μL of the resazurin (0.15% w/v) was added to per well. The microplate was incubated at 36°C for 4 h, and the growth of microorganisms was interpreted by following the change in well colors.

### 2.4 Anticancer activity

#### 2.4.1 Cell culture

This study included three epithelial cancerous (AR42J, pancreatic cancer; HeLa, cervical cancer, and MDA‐MB‐231 breast cancer) and two noncancerous epithelial human cells (ARPE‐19, retina; and MCF10A, breast). AR42J (ECACC, Cat No. 93100618) and MDA‐MB‐231 (ATCC, Cat No. HTB‐26) cells were cultured in RPMI media. HeLa cells (ATCC, Cat No. CCl‐2) were cultured in EMEM media. ARPE‐19 (ATCC, Cat No. CRL‐2302) and MCF10A (ATCC, Cat No. CRL‐10317) cells were cultured in DMEM. Each medium consisted of penicillin–streptomycin (1%) and fetal bovine serum (10%). Cells were cultured in a 37°C incubator with 5% CO_2_ humidification. Cells were cultured in 96‐well plates as 10.000 cells per well. After they reached at 80% confluency, cells were treated with extracts. For this, experiments for each cell included two groups, (1) treated with *T. coelesyriacus* extracts obtained by methanol for 24 h, 48 h, and 72 h at 3.125, 6.25, 12.5, 25, 59, 100, and 200 μg/mL, and (2) treated with *T. coelesyriacus* extracts obtained by water for 24 h, 48 h, and 72 h at 3.125, 6.25, 12.5, 25, 59, 100, and 200 μg/mL. Control wells for each cell were untreated (0 μg/mL). Positive control experiments were performed with cisplatin at the same concentrations with extracts (3.125–100 μg/mL) for 24 h, 48 h, and 72 h. For each dose at each concentration, at least three wells were used as intra‐experimental repeats. The whole experiments for each cell were repeated as two to three independent replications.

#### 2.4.2 MTT cytotoxicity assay

After treatment with different extracts (at 3.125, 6.25, 12.5, 25, 50, 100, and 200 μg/mL for 24 h, 48 h, and 72 h), media with extracts were removed, and the MTT protocol was applied as before (Cakmak et al., 2021). MTT was also applied after cisplatin treatment for 24 h, 48 h, and 72 h. The concentrations of cisplatin used were 3.125, 6.25, 12.5, 25, 50, and 100 μg/mL. In this method, 200 μL of media, including 10 μL MTT (3‐(4,5‐dimethylthiazol‐2‐yl)‐2,5‐diphenyltetrazolium bromide), was added to each well and incubated at 37°C for 2.5 h. After incubation, media with MTT were removed, and 150 μL of DMSO was added to each well for dissolving formazan crystals. Subsequently, plates were incubated in the dark on a shaker for 1 h. Absorbances at 570 nm were read. Cell viabilities (as percentages) were calculated according to the viability of untreated cells considered as 100%. If applicable, IC50 values were calculated as before (Uzuner et al., 2021).

### 2.5 Molecular docking

Molecular docking studies were conducted using the AutoDock Vina 1.5.7 program (Eberhardt et al., 2021). 3D structures of *S. aureus* MurB (PDB ID: 1HSK) and human FYN kinase (PDB ID: 2DQ7) as target protein structures were retrieved from the Protein Data Bank (PDB). Water molecules and any bound ligand were excluded from target protein 3D structures. However, flavin adenine dinucleotide (FAD), the major cofactor of the MurB protein bound to the 1HSK active site, was retained during MurB‐related molecular docking studies. Both target protein structures were monomerized, and the protonation states were counted by PROPKA 3.0 at pH = 7.0 (Olsson et al., 2011). All amino acids were charged accordingly. The pdb files of natural ligands were built using Open Babel (O'Boyle et al., 2011). The pdbqt file formats of both target proteins and extracted ligand compounds were prepared by employing AutoDock tools 1.5.7.

Polar hydrogens were added to the 3D structures of both MurB and FYN, and Kollman charges were also calculated. Gasteiger charges were calculated for natural compounds as ligands, and pdbqt files were saved. Each docking assay was applied on both MurB and FYN protein targets via three independent runs using ligand pdbqt files. The grid dimensions for 1HSK were set as −30 Å × 25 Å × 30 Å and grid center coordinates were adjusted as x = 178 Å, y = 155 Å, z = 155 Å, respectively, whereas 2DQ7 docking coordinates were set as −19 Å × 20 Å × −15 Å for grid dimensions and x = 25 Å, y = 35 Å, z = 25 Å for grid center coordinates. The docking outcomes for each compound were investigated in detail, and specific 2D interaction maps were analyzed using the Discovery Studio Accelrys (Discovery Studio Visualizer v21.1.0.20298) program (Dassault Systems, BIOVIA).

### 2.6 Statistical analysis

For statistical analyses, version 13 of SPSS program was used. Cell viabilities (%) calculated from MTT assay were statistically compared using UNIANOVA test. *p* values less than .05 were considered as significant. The significance levels used are *p* < .05 (*), *p* < .01 (**), *p* < .001 (***), and *p* < .0001 (****). Experiments included inter‐ and intrarepeats, and standard errors of the mean (± S.E.M.) were shown by error bars in the graphs.

## 3 RESULTS

### 3.1 Analysis of the extracts using LC‐HRMS

The prepared plant extracts were analyzed with LC‐HRMS to determine their content. The LC‐HRMS analysis of *T. coelesyriacus*, which has the highest total phenolic content, was carried out due to the spectrophotometric study. It identified 12 chemicals as being present. Table 1 displays the retention time (Rt), the amount of each chemical, and the discovered molecular ion. In *T. coelesyriacus*, 25.12 g/kg of individual phenolic compounds was discovered in methanol extract, and 13.03 g/kg of individual phenolic compounds was discovered in water extract. The most prevalent compound in methanol extract was chlorogenic acid, which had a mass of 13.58 g/kg, followed by quinic acid, 7.81 g/kg, and vitexin, 1.41 g/kg. Additionally, the most prevalent compound in water extract was quinic acid, which had a mass of 8.79 g/kg, followed by epicatechin, 1.36 g/kg.

**TABLE 1:** Selected retention times, *m*/*z* ratios, and quantitative content of water and methanol extracts in the UHPLC‐ESI‐MS/MS method for the phenolic compounds.

Compounds	Found *m*/*z* (molecular ion)	Rt (min)	Water extract	Methanol extract
			Amount of phenolic comp. g/kg	Amount of phenolic comp. g/kg
Fumaric acid	114.9	4.032	0.5916	0.5350
Chlorogenic acid	352.9	10.824	0.9763	13.5768
Keracyanin chloride	592.8	9.892	0.0061	0.1100
Quinic acid	190.9	2.477	8.7850	7.8116
Gallic acid	168.9	5.567	0.2541	0.0721
Cyanidin‐3‐*O*‐glucoside	447.1	10.372	0.0991	0.2877
Epicatechin	289.0	11.508	1.3567	0.6443
Vitexin	430.9	11.548	0.9293	1.4102
Naringin	579.0	11.952	–	0.0037
Ferulic acid	193.0	12.542	–	0.0210
Rosmarinic acid	358.8	12.462	0.0286	0.4425
Luteolin	284.9	13.409	–	0.2067

### 3.2 Antibacterial and antifungal activities

#### 3.2.1 Results of agar dilution method

In the agar dilution method, the antibacterial activity results of the methanol extract of *T. coelesyriacus* against *S. aureus*, *E. aerogenes*, *E. coli*, *P. aeruginosa*, and *K. pneumonia* are demonstrated in Figure 1. After incubation at 36°C for 24 h, the plates of 0.42, 0.21, 0.1 mg/mL and the control (0 mg/mL) methanol extract of *T. coelesyriacus* against *S. aureus*; plates, J, K, L, and I (a few colonies) were covered with pale yellow bacterial colonies, respectively. No colonies were detected on plates A, B, C, D, E, F, G, and H treated with 106.6, 53.3, 26.6, 13.3, 6.6, 3.3, 1.67, and 0.83 mg/mL methanol extract of *T. coelesyriacus*, respectively. Therefore, the MIC value of the methanol extract of *T. coelesyriacus* against *S. aureus* was found to be 0.83 mg/mL. Colonies began to seem from the C‐plate, where 26.6 mg/mL methanol extract of *T. coelesyriacus* concentration was used for *E. coli* and *E. aerogenes*. Hence, this experiment determined the MIC value of *T. coelesyriacus* methanol extract against *E. aerogenes* and *E. coli* to be 53.3 mg/mL. *K. pneumonia* and *P. aeruginosa* colonies were evident from the H‐plate, where 0.83 mg/mL methanol extract of *T. coelesyriacus* concentration was used. Therefore, the MIC value of the methanol extract of *T. coelesyriacus* against *K. pneumonia* and *P. aeruginosa* was found to be 1.67 mg/mL. (Figure 1).

**FIGURE 2:**
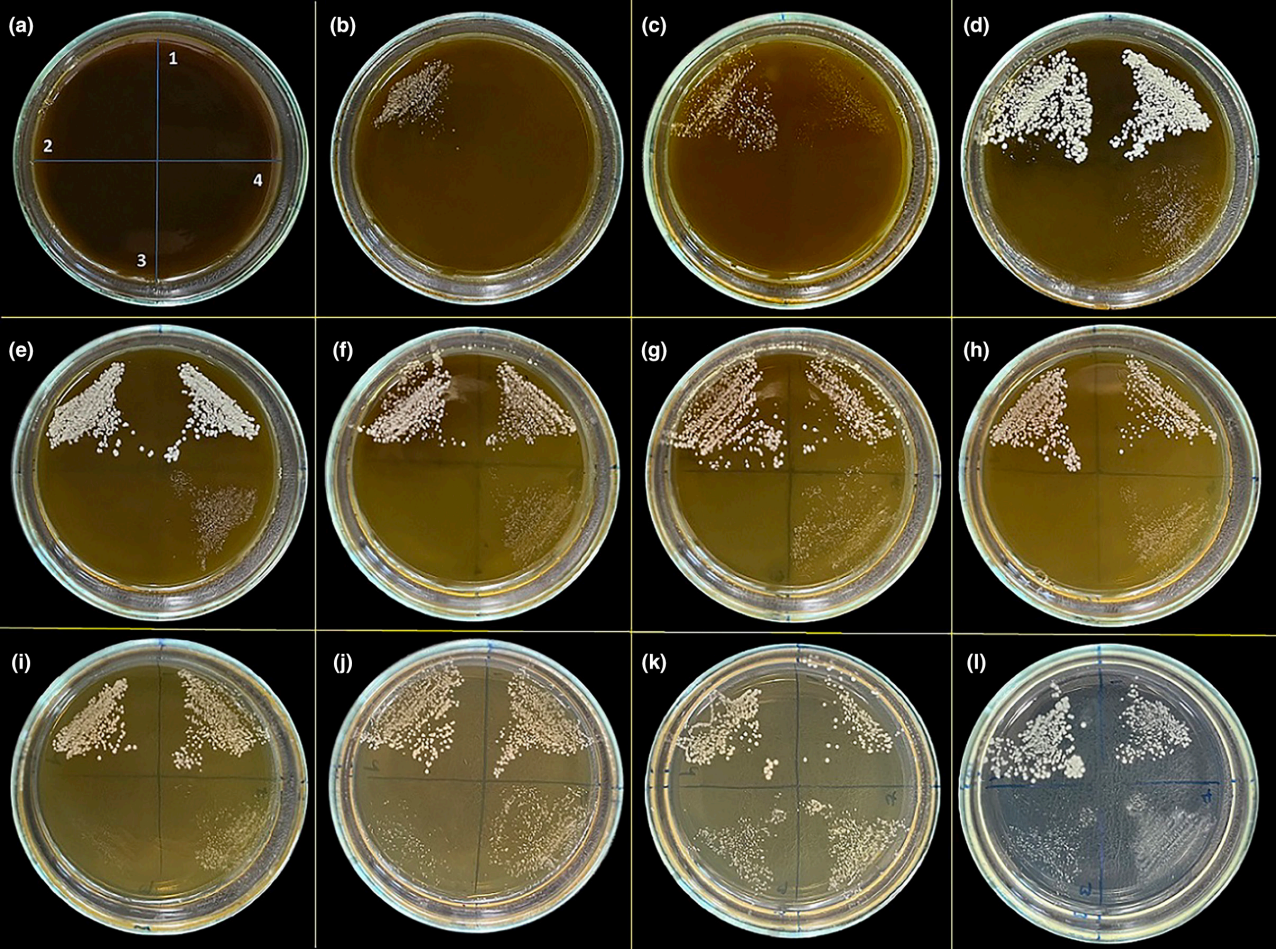
Petri dish pictures of the antifungal activity assay of the *T. coelesyriacus* methanol extract with different concentrations against four yeast strains: (1) *C. albicans*, (2) *C. tropicalis*, (3) *C. krusei*, and (4) *C. parapsilosis*. (a) 106.6 (b) 53.3, (c) 26.6, (d) 13.3, (e) 6.6, (f) 3.3, (g) 1.67, (h) 0.83, (i) 0.42, (j) 0.21, (k) 0.1 mg/mL methanol extract of *T. coelesyriacus*, (l) Control.

The antifungal activity results of *T. coelesyriacus* methanol extract against *C. albicans*, *C. tropicalis*, *C. krusei*, and *C. parapsilosis* are demonstrated in Figure 2. After incubation at 36°C for 24 h, *C. albicans* colonies began appearing from the C‐plate, where 26.6 mg/mL of methanol extract concentration was used. So, the MIC value of the methanol extract of *T. coelesyriacus* against *C. albicans* was found to be 53.3 mg/mL. *C. tropicalis*, *C. krusei*, and *C. parapsilosis* colonies began appearing from the B‐plate, I‐plate, and D‐plate, respectively. Therefore, the MIC values against *C. tropicalis*, *C. krusei*, and *C. parapsilosis* were 106.6, 0.83, and 26.6 mg/mL, respectively. In antibacterial and antifungal studies with water extract of *T. coelesyriacus* against *S. aureus*, *E. aerogenes*, *E. coli*, *K. pneumonia*, *P. aeruginosa*, *C. albicans*, *C. tropicalis*, *C. krusei*, and *C. parapsilosis*, it was observed that water extract of *T. coelesyriacus* did not have any inhibitory effect on the bacterial and yeast strains.

#### 3.2.2 Results of broth microdilution method

**FIGURE 3:**
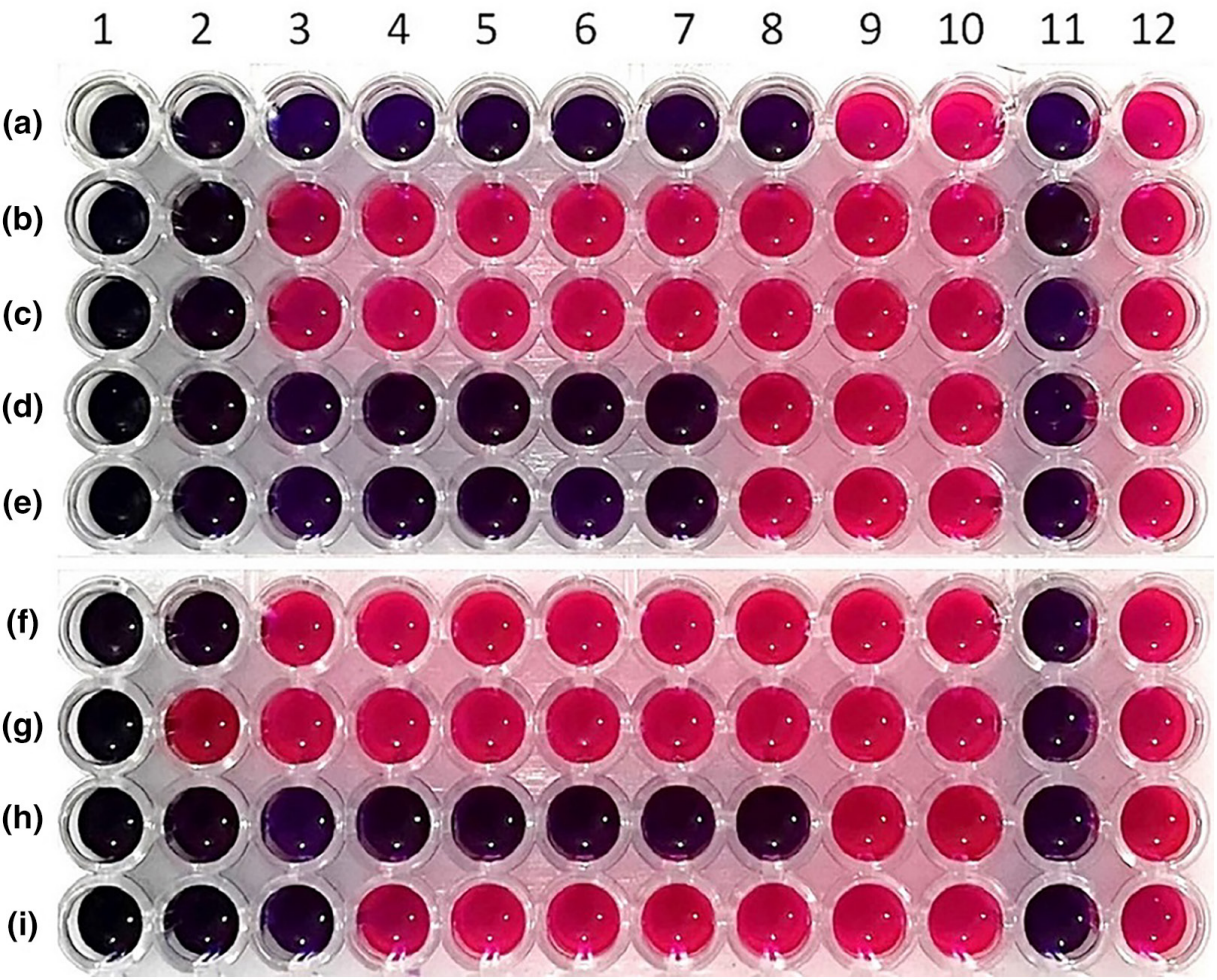
Microplate picture of the methanol extract of *T*. *coelesyriacus* antibacterial and antifungal activities test against *S. aureus* (a), *E. aerogenes* (b), *E. coli* (c), *P. aeruginosa* (d), *K. pneumonia* (e), *C. albicans* (f), *C. tropicalis* (g), *C. krusei* (h), and *C. parapsilosis* (i). The methanol extract concentration in wells A8 (0.83 mg/mL), B2 (53.3 mg/mL), C2 (53.3 mg/mL), D7 (1.67 mg/mL), E7 (1.67 mg/mL), F2 (53.3 mg/mL), G1 (106.6 mg/mL), H8 (0.83 mg/mL), and I3 (26.6 mg/mL) with no color change were determined as MIC values against *S. aureus*, *E. aerogenes*, *E. coli*, *P. aeruginosa*, *K. pneumonia*, *C. albicans*, *C. tropicalis*, *C. krusei*, and *C. parapsilosis*, respectively. 11. negative control, 12. positive control.

The antibacterial and antifungal activities of the methanol extract of *T. coelesyriacus* against *S. aureus, E. aerogenes, E. coli, P. aeruginosa, K. pneumonia, C. albicans, C. tropicalis, C, krusei and C. parapsilosis* was repeated using the broth dilution assay. Methanol extract of *T. coelesyriacus* was added to wells 1–10 on the microplate at concentrations of 21.33, 10.66, 5.33, 2.66, 1.33, 0.66, 0.33, 0.16, 0.08, and 0.04 mg/mL, respectively. The negative control is the 11th well, which confirms no contamination has occurred during the experiment. The positive control is the 12th well, representing the viability of the microbial strains. The MIC value is determined as the methanol extract of *T. coelesyriacus* concentration with the least amount of the extract on the microplate with no change in color. Pink indicates microorganism growth and, thus viability, while blue indicates growth inhibition (Elshikh et al., 2016; Unver & Gurhan, 2024). As a result of this experiment, same as the agar dilution test results, the MIC values of the methanol extract against bacterial strains *S. aureus*, *E. coli*, *E. aerogenes*, *P. aeruginosa*, and *K. pneumonia* were determined as 0.83, 53.3, 53.3, 1.67 and 1.67 mg/mL respectively, in the 9th, 2nd, 2nd, 7th and 7th wells without color change. The MIC values of the methanol extract against yeast strains *C. albicans*, *C. tropicalis*, *C. krusei* and *C. parapsilosis were found to be* 53.3, 106.6, 0.83, and 26.6 mg/mL, respectively, in the 2nd, 1st, 8th and 4th wells with no color change (Figure 3, Table 2). The antifungal and antibacterial activity of the water extract of *T. coelesyriacus* was repeated using a broth dilution assay. However, it was determined that the water extract of *T. coelesyriacus* had no inhibitory effect on the bacterial and yeast strains.

**TABLE 2:** MIC values of the methanol extract of *T. coelesyriacus* against different microorganisms.

Microorganisms	Methanol extract of *T. coelesyriacus* (mg/mL)
*S. aureus*	0.83
*E. aerogenes*	53.3
*E. coli*	53.3
*P. aeruginosa*	1.67
*K. pneumonia*	1.67
*C. albicans*	53.3
*C. tropicalis*	106.6
*C. krusei*	0.83
*C. parapsilosis*	26.6

#### 3.2.3 Cytotoxic profile in human cancerous and normal cells

**FIGURE 4:**
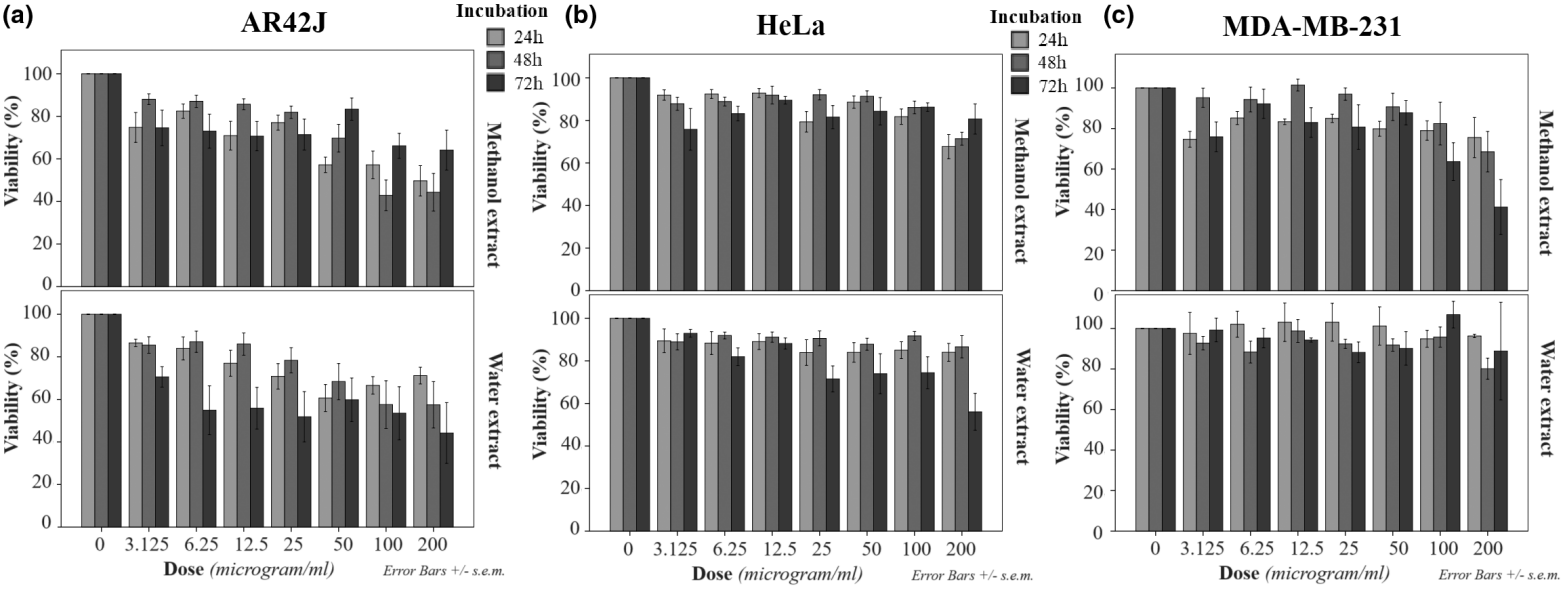
Cell viabilities (%) in AR42J (pancreatic cancer) cells (a), HeLa (cervical cancer) cells (b), and MBD‐MB‐231 (breast cancer) (c) cells after the treatments for 24 h, 48 h, or 72 h with *T. coelesyriacus* extracts obtained either by methanol (up panels) or by water (down panels) at different concentrations.

The treatment with the maximum dose (200 μg/mL) of methanol extract of *T. coelesyriacus* for 24 h and 48 h resulted in the death of almost half of AR42J but not at 72 h (Figure 4a, up panel), while the viability of MDA‐MB‐231 cell population decreased at half after the maximum dose for 72 h only (Figure 4c, up panel). However, HeLa cells were the most resistant to methanol extract as 70% of cells were still viable after the maximum dose for the extended incubation till 72 h (Figure 4b, up panel). AR42J was also more responsive to lower doses (50 μg/mL for 24 h and 100 μg/mL for 48 h) (Figure 4a, up panel) compared to HeLa and MDA‐MB‐231 cells.

In the experimental groups for the treatments with water‐extracted *T. coelesyriacus*, AR42J was the more responsive cancer cell to the lower doses from 6.25 to 200 μg/mL for 72 h as half of AR42J died (Figure 4a, down panel). At lower incubations (24 h and 48 h), the cell death rate in AR42J was higher than other cells at the same doses of water extracts (Figure 4b,c, down panels). Fifty percent of HeLa cells died at 200 μg/mL for 72 h (Figure 4b, down panel). However, MDA‐MB‐231 cells were the most resistant cell type to water extract of *T. coelesyriacus* (Figure 4c, down panel).

Statistical analyses for viability in Figure 4 are summarized for each cell in Table 3. According to the UNIANOVA test, the dose itself was significant in AR42J, HeLa, and MDA‐MB‐231 cells with *p* values as 0 (****). Extraction type was significant in only MDA‐MB‐231 cells (*p* < .0001) but not in other cells as both extracts were cytotoxic in AR42J, and the extraction method did not reveal a significant difference in HeLa cells. However, incubation itself was not significant in MDA‐MB‐231 cells but significant in HeLa (*p* < .0001) and AR42J (*p* < .01) cells. The synergistic effect of dose and incubation were significant in AR42J cells only (*p* < .001), while extract and incubation together significantly affected cell viability in AR42J, HeLa, and MDA‐MB‐231 cells with *p* values less than .0001 (****), .5 (*), and .001 (***), respectively. The total effect of dose, incubation, and extract was significant in HeLa cells only. Calculated IC50 values representing the cytotoxic dose for half of the cell population are shown in Table 4. The minimum IC50 values were detected for (1) 24‐h incubation with methanol extract in AR42J cells (average: 77.31), (2) 72‐h incubation with water extract in AR42J cells (average: 75.04), and (3) 72‐h incubation with methanol extract in MDA‐MB‐231 cells (average: 76.69). The less IC50 means, the higher the cytotoxic effect. In normal cells, IC50 values could not be detected because there were no conditions that 50% of cell death occurred.

**TABLE 3:** Summary of statistical comparisons for the cells in Figure 4.

Cells	AR42J	HeLa	MDA‐MB‐231
Comparison	Significance (*p* values)		
Dose	.000****	.000****	.000****
Extract type	.723	.919	.000****
Incubation	.002**	.000****	.084
Dose and extract	.461	.183	.016*
Dose and incubation	.001***	.258	.691
Extract and incubation	.000****	.016*	.002**
Dose and extract and incubation	.342	.016*	.473

* *p* < .05.

** *p* < .01.

*** *p* < .001.

**** *p* < .0001.

**TABLE 4:** IC50 values for in vitro cytotoxicity of *T*. *coelesyriacus* in human cancers.

Cell	Extract	Incubation	IC50 value (μg/mL)
AR42J	Methanol	24 h	77.31 ± 18.58
		48 h	86.94 ± 20.04
	Water	48 h	92.02 ± 26.38
		72 h	75.04 ± 14.07
HeLa	Water	72 h	91.30 ± 18.10
MDA‐MB‐231	Methanol	72 h	76.69 ± 2.11

**FIGURE 5:**
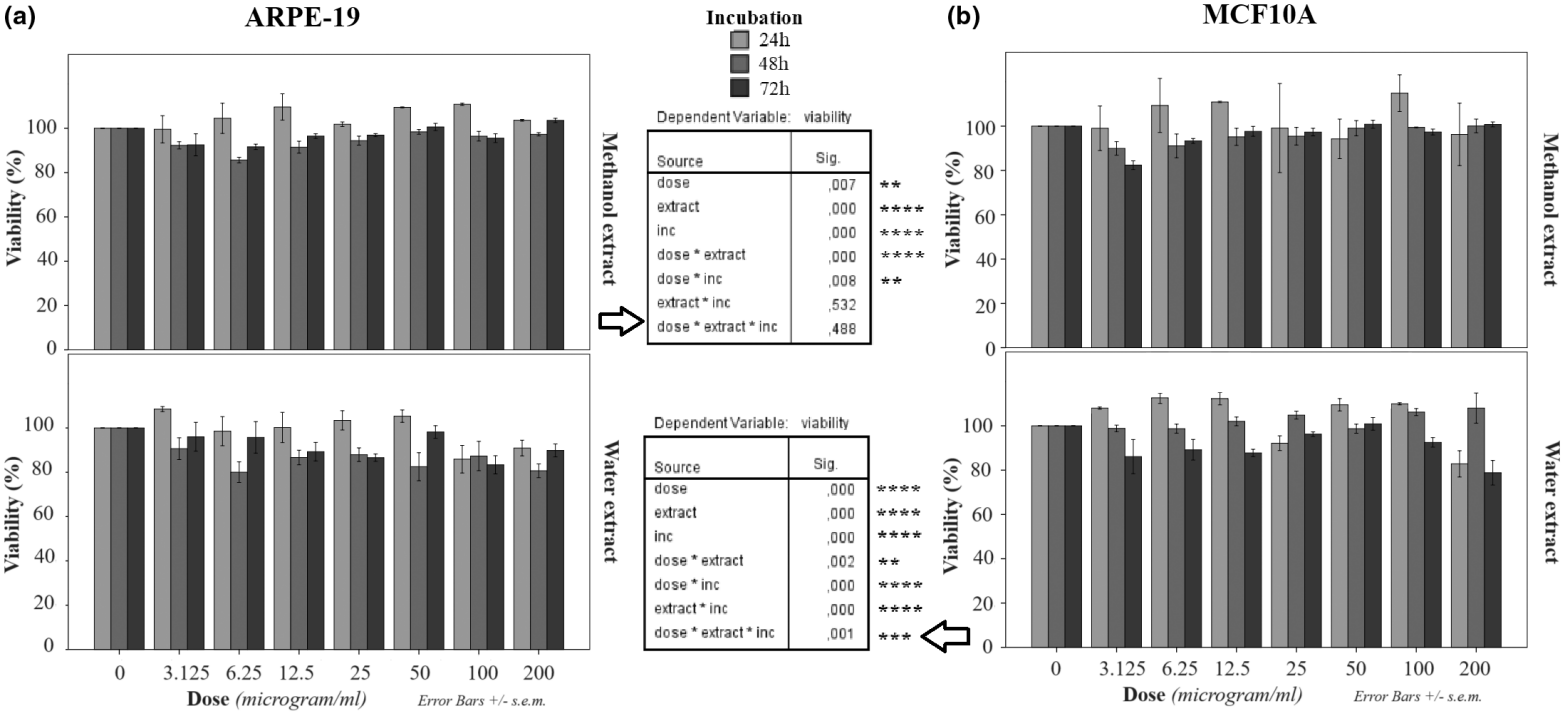
Cell viabilities (%) in ARPE‐19 (retinal epithelial) cells (a) and MCF10A (breast epithelial) cells (b) after the treatments for 24 h, 48 h, or 72 h with *T. coelesyriacus* extracts obtained either by methanol (up panels) or by water (down panels) at different concentrations. Related statistics are given for each graph. *p* < .05 (*), *p* < .01 (**), *p* < .001 (***), and *p* < .0001 (****).

The same experiments were performed for noncancerous healthy cells including ARPE‐19 and MCF10A (Figure 5a,b). The combined effect of dose, extract type, and incubation was insignificant in ARPE‐19 (Figure 5a) but significant in MCF10A cells (***) (Figure 5b). Each nominal parameter, including dose, incubation, and extract, was individually significant in both cells. However, the minimum cell viability (around 80%) was detected in normal cells after the treatment with water extraction when compared to cancer cells. In general, the cell viability of normal cells was significantly higher than that of cancerous cells. Therefore, IC50 values for normal cells could not be detectable.

We then performed MTT assay for cisplatin treatment for 24 h, 48 h and 72 h in five cell lines examined in this study (Table 5). IC50 values for cisplatin are less than IC50 values detected after *T. coelesyriacus* extracts. IC50 values gradually decreased in all cells (except HeLa with some fluctuations) for extended incubations indicating that cisplatin at lower concentrations can provoke cell death for longer incubations. However, cisplatin also induced cell death in normal cells as well as cancerous cells.

**TABLE 5:** IC50 values for in vitro cytotoxicity of a standard drug, cisplatin, in cancerous and normal cells.

Cell type		Ave IC50 values (μg/mL)		
		24 h	48 h	72 h
Cancer cells	AR42J	18.94	14.84	6.18
	HeLa	8.16	13.92	4.56
	MDA‐MB‐231	41.28	27.87	10.92
Normal cells	MCF10A	33.56	26.03	8.32
	ARPE19	63.42	54.14	16.01

### 3.3 Molecular docking data

The initial stage of peptidoglycan synthesis in *S. aureus* involves the conversion of phosphoenolpyruvate and UDP‐*N*‐acetylglucosamine to UDP‐*N*‐acetylenolpyruvylglucosamine (UDP‐GlcNAcEP)3 by MurA. Afterwards, UDP‐GlcNAcEP is reduced to UDP‐*N*‐acetylmuramic acid by MurB. Then, MurC, MurD, MurE, and MurF enzymes add five amino acid residues (L‐Lys, L‐Ala, D‐Ala‐D‐Ala, and D‐Glu) sequentially to the D‐lactyl group of UDP‐*N*‐acetylmuramic acid in a ribosome‐independent manner. Since MurB is a crucial enzyme in the peptidoglycan synthesis of *S. aureus* (Matsuo et al., 2003), chemical compounds that hinder MurB activity are considered as potential antimicrobial agents (Nishida et al., 2006). Most of the native compounds of *T. coelesyriacus* were energetically preferred for the *S. aureus* MurB active site. Of these, keracyanin chloride, naringin, and vitexin were determined as the leading MurB inhibitory compounds of *T. coelesyriacus* in the presence of low binding free energy values: −9.4, 9.2, and 8.2 kcal/mol, respectively (Table 6).

**TABLE 6:** Molecular docking data and interacting residue profiles of *T. coelesyriacus* native compounds when directed to *S. aureus* MurB enzyme.

Compound id	Binding energy 1HSK (kcal/mol)	Interacting residues	H bonds
Chlorogenic acid	−7.2	**Chain A**: ALA154 TYR155 GLY156 TYR187 ARG225 LYS228 GLN229 SER238 PHE240 GLN241 ARG242 PRO243 PHE247 ALA248 GLY249 HIS271 PHE274	4
Cyanidin‐3‐*O*‐glucoside	−7.9	**Chain A**: GLY153 ALA154 TYR155 TYR187 ARG188 ARG225 GLN229 SER238 PHE240 GLN241 ARG242 PHE247 ALA248 GLY249 LYS250 HIS271 GLY273 GLU308	5
Epicatechin	−7.1	**Chain A**: GLY153 ALA154 TYR155 ARG225 GLN229 SER238 ARG242 PHE247 ALA248 GLY249 LYS250 HIS271 ALA272 GLY273 PHE274	3
Ferulic acid	−6.2	**Chain A**: LEU17 VAL25 ALA37 LYS39 MET58 VAL67 ILE80 THR82 GLU83 TYR84 MET85 ASN86 GLY88 SER89 LEU137 ALA147	4
Fumaric acid	−4.3	**Chain A**: VAL25 ALA37 ILE38 LYS39 GLU54 MET58 VAL67 ILE80 THR82 GLU83 LEU137 ALA147 ASP148 PHE149	4
Gallic acid	−5.8	**Chain A**: PHE22 LYS39 MET46 SER50 PHE51 LEU52 GLU53 GLU54 ALA55 ASP148 PHE149 GLY150 LEU151 ARG153	2
Keracyanin chloride	−9.4	**Chain A**: ASN83 MET150 GLY153 ALA154 TYR155 TYR187 ARG188 ARG225 GLN229 GLY237 SER238 PHE240 GLN241 ARG242 PHE247 ALA248 GLY249 LYS250 HIS271 PHE274 GLU308	6
Luteolin	−7.7	**Chain A**: ASN83 MET150 ALA154 TYR155 TYR187 ARG188 ARG225 GLN229 SER238 PHE240 ARG242 PHE274 GLU308	7
Naringin	−9.2	**Chain A**: GLY153 ALA154 TYR155 GLY156 GLY157 GLU158 TYR187 ARG188 ARG224 ARG225 LYS228 GLN229 SER238 PHE240 GLN241 ARG242 ALA248 HIS271 PHE274 GLU308	6
Quinic acid	−5.6	**Chain A** LEU17 VAL25 ALA37 ILE38 LYS39 GLU54 MET58 VAL67 ILE80 THR82 GLU83 TYR84 MET85 LEU137 ALA147 ASP148 PHE149	4
Rosmarinic acid	−7.3	**Chain A**: ASN83 MET150 TYR187 ARG188 GLY237 SER238 PHE240 GLN241 ARG242 PRO243 HIS246 PHE247 ALA248 GLY249 LYS250 HIS271 GLU308	7
Vitexin	−8.2	**Chain A**: GLY153 ALA154 TYR155 TYR187 ARG188 ARG225 GLN229 SER238 PHE240 GLN241 ARG242 PHE247 ALA248 GLY249 LYS250 HIS271 PHE274 GLU308	5

**FIGURE 6:**
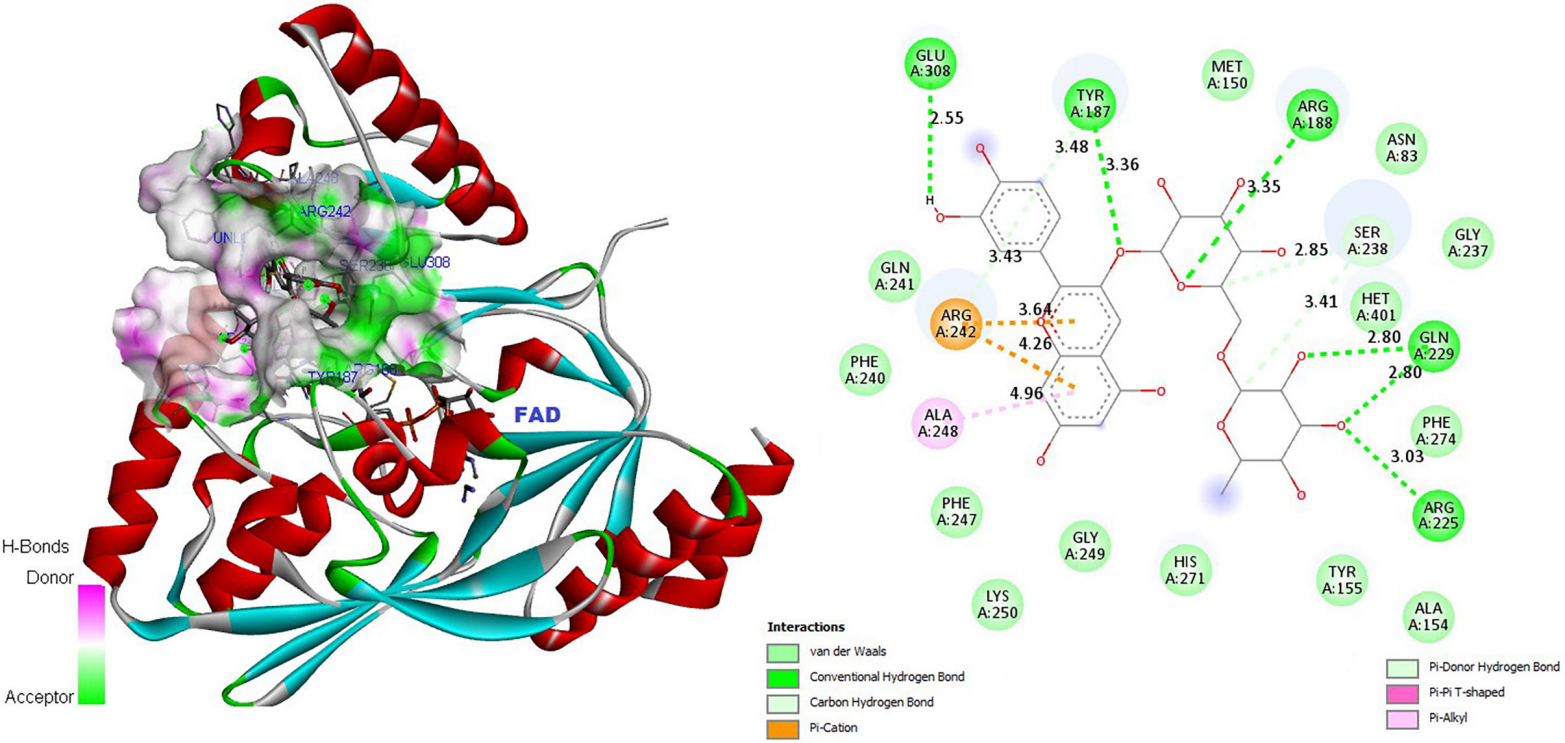
Molecular docking pose of keracyanin chloride‐MurB (1HSK) and 2D depiction of their biochemical interaction potential. Green dashed lines represent potential hydrogen (H) bonds between keracyanin chloride and 1HSK catalytic residues. The calculated short H bond lengths prove the close proximity and high biochemical interaction potential between keracyanin chloride and 1HSK catalytic residues.

**FIGURE 7:**
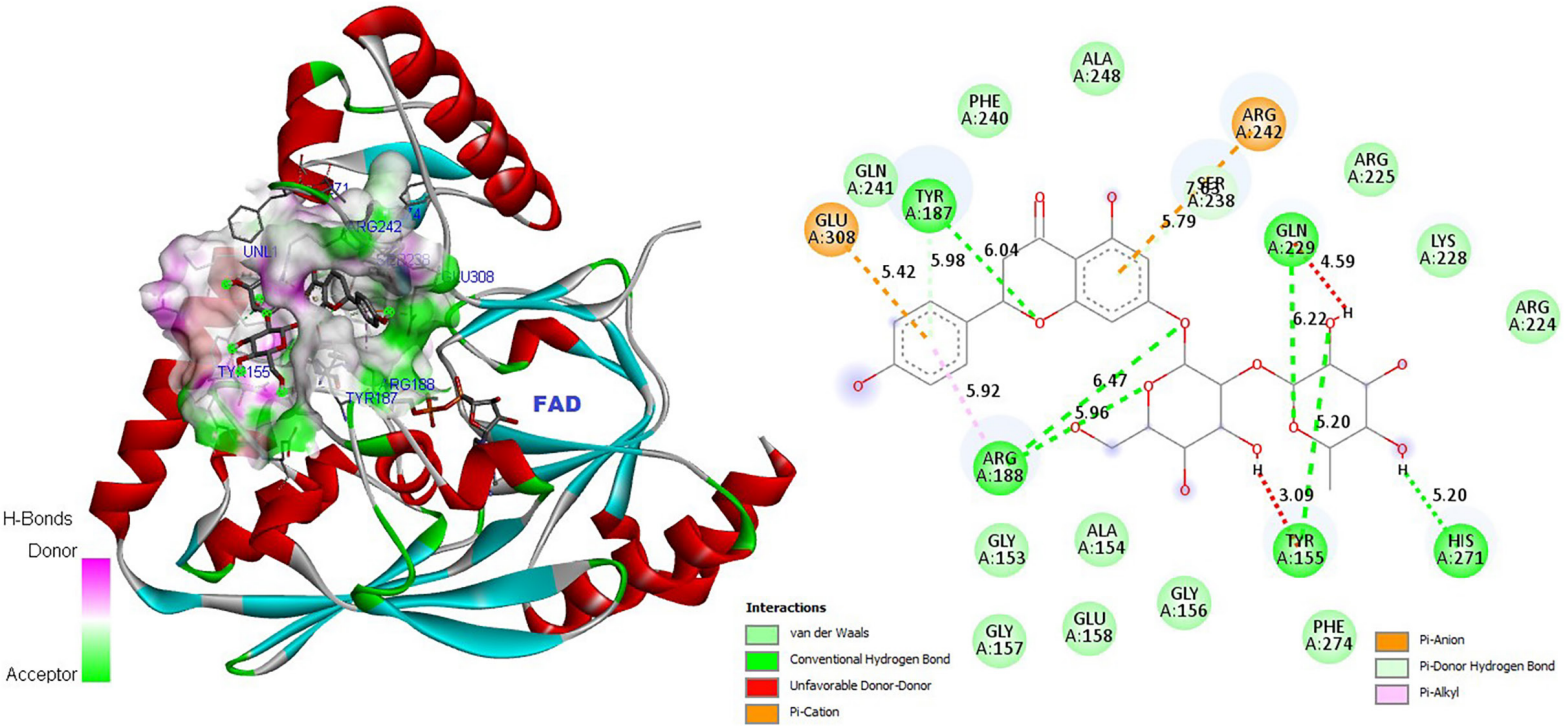
Molecular docking pose of naringin‐MurB (1HSK) and 2D depiction of their biochemical interaction potential. Green dashed lines represent potential H bonds between naringin and 1HSK catalytic residues.

**FIGURE 8:**
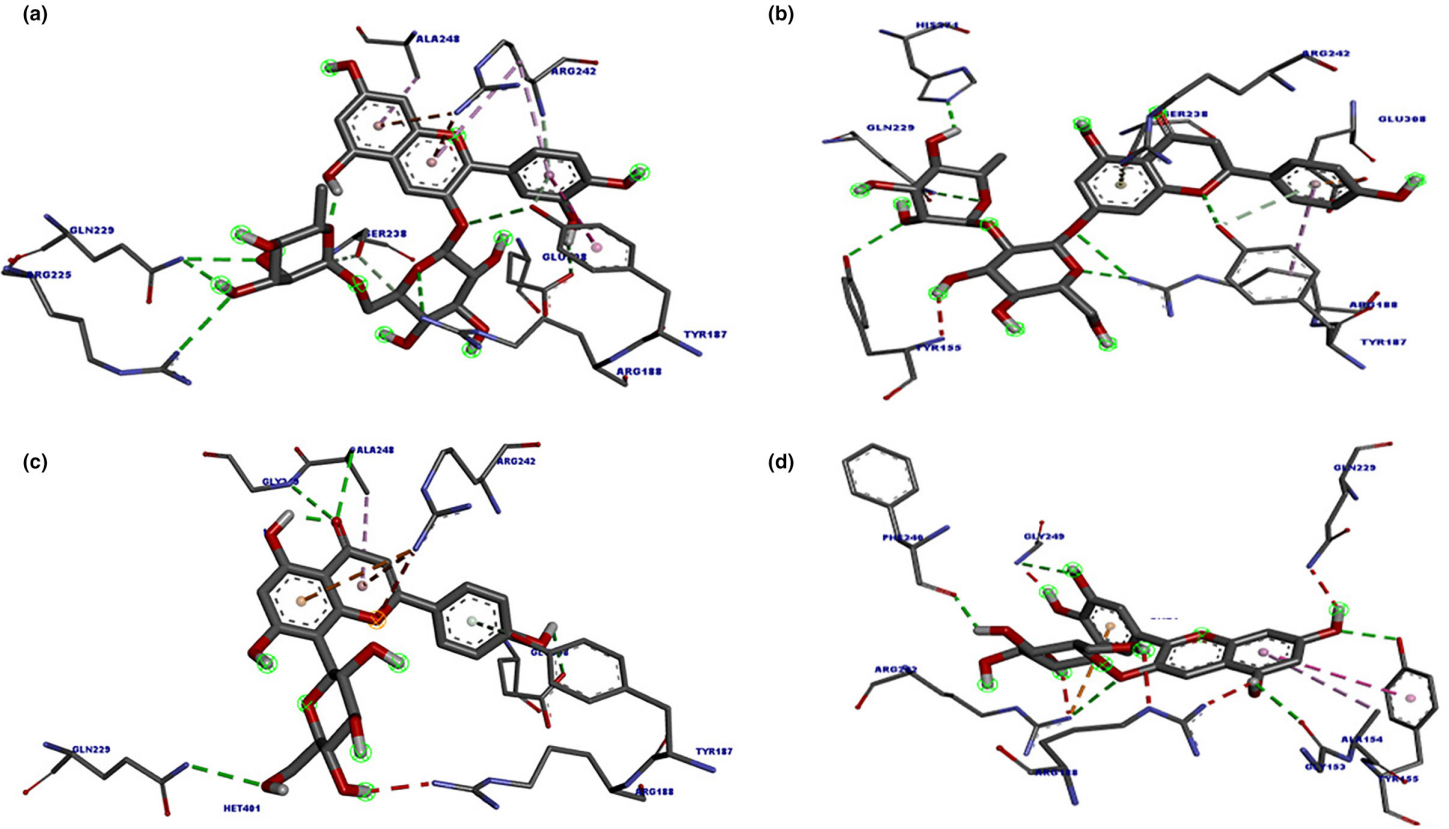
Binding pose predictions for the four ligands with the lowest ∆G values superimposed on the crystallographic structure of 1HSK target. (a) Keracyanin chloride, (b) Naringin, (c) Vitexin, (d) Cyanidin‐3‐*O*‐glucoside. Key hydrogen bonds are shown by green lines, and the protein molecular surface is colored by atom type. Each pose was analyzed via BIOVIA Discovery Studio Visualizer.

In‐depth analysis of the keracyanin chloride binding mode to the catalytic core of the *S. aureus* MurB protein revealed that it is capable of forming six hydrogen (H) bonds with the 1HSK active site residues (Figure 6). Similarly, as an indicator of strong binding, naringin has the potential to form a total of six H bonds with 1HSK catalytic residues (Figure 7). Potential binding pose predictions for four ligands showing high affinity for the 1HSK target in the presence of the lowest ∆G values were also examined (Figure 8a–d). Furthermore, rosmarinic acid and luteolin, the two other natural compounds of *T. coelesyriacus*, were able to form seven H bonds within the catalytic core of MurB, suggesting their potential antibacterial activity against *S. aureus* pathogenic bacteria.

**FIGURE 9:**
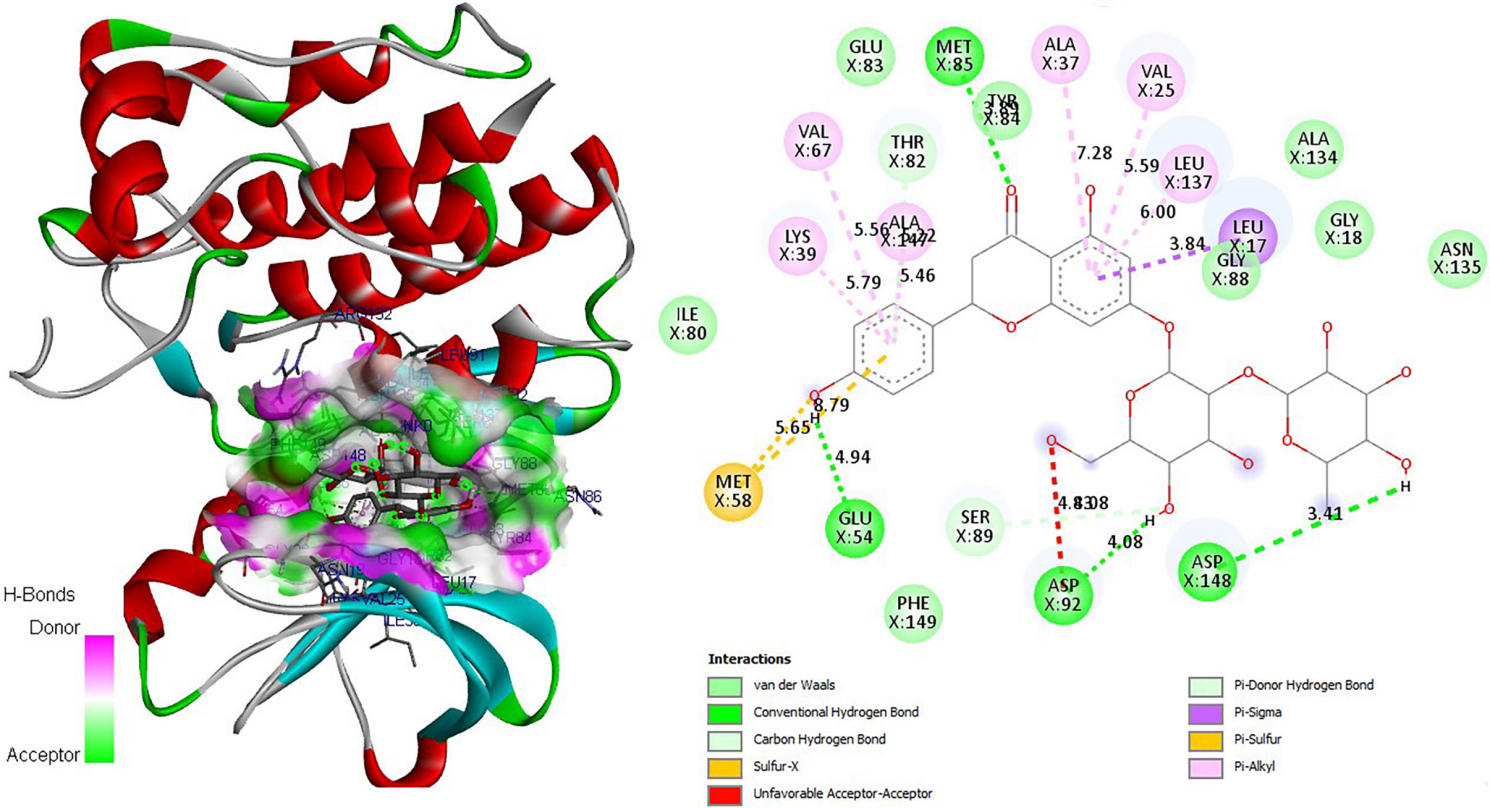
Molecular docking pose of naringin‐FYN (2DQ7) and 2D depiction of their biochemical interaction potential. Green dashed lines denote potential H bonds between naringin and 2DQ7 catalytic residues.

**FIGURE 10:**
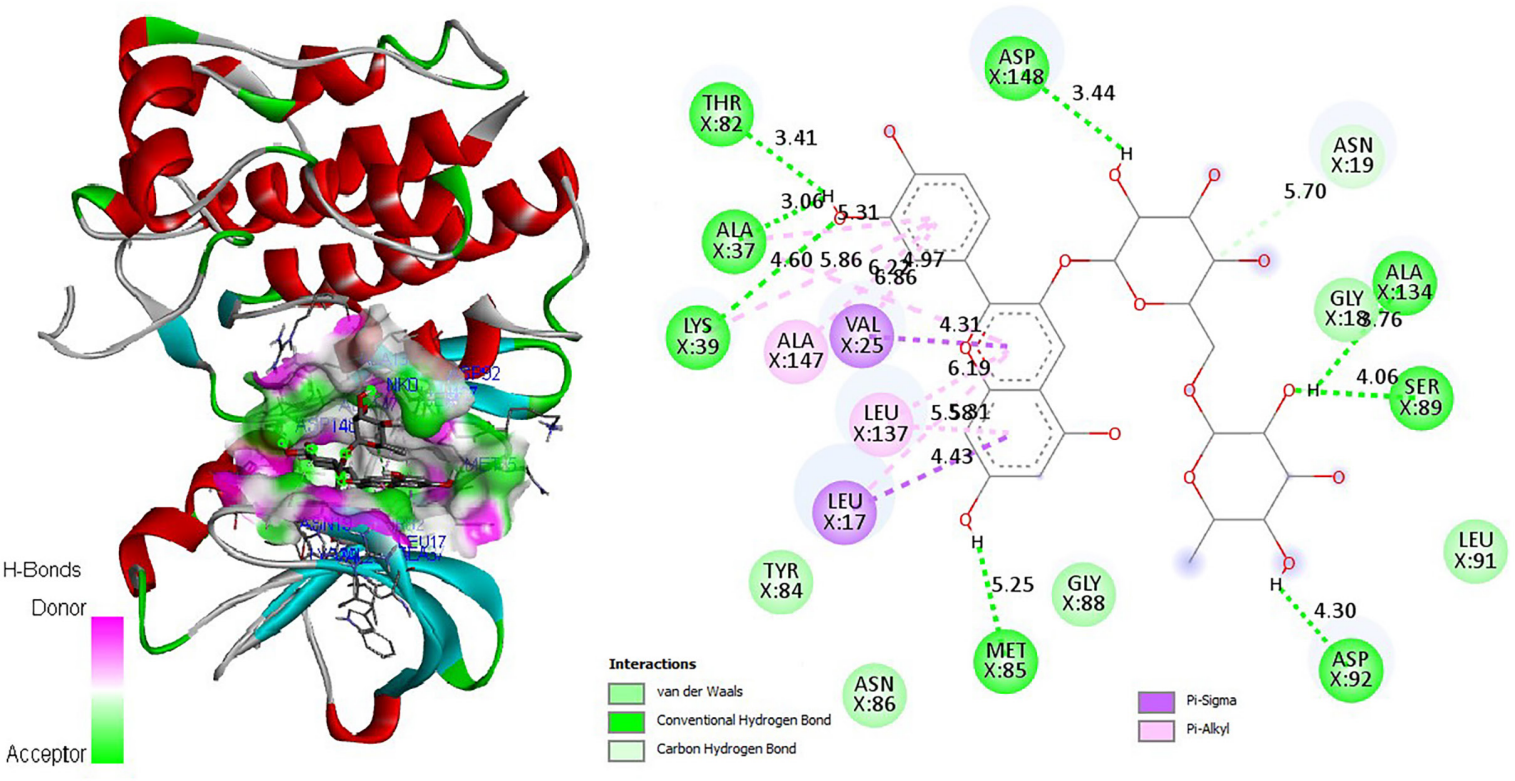
Molecular docking pose of keracyanin chloride‐FYN (2DQ7) and 2D depiction of their biochemical interaction potential. Green dashed lines represent potential H bonds between keracyanin chloride and the catalytic residues of FYN kinase protein.

**FIGURE 11:**
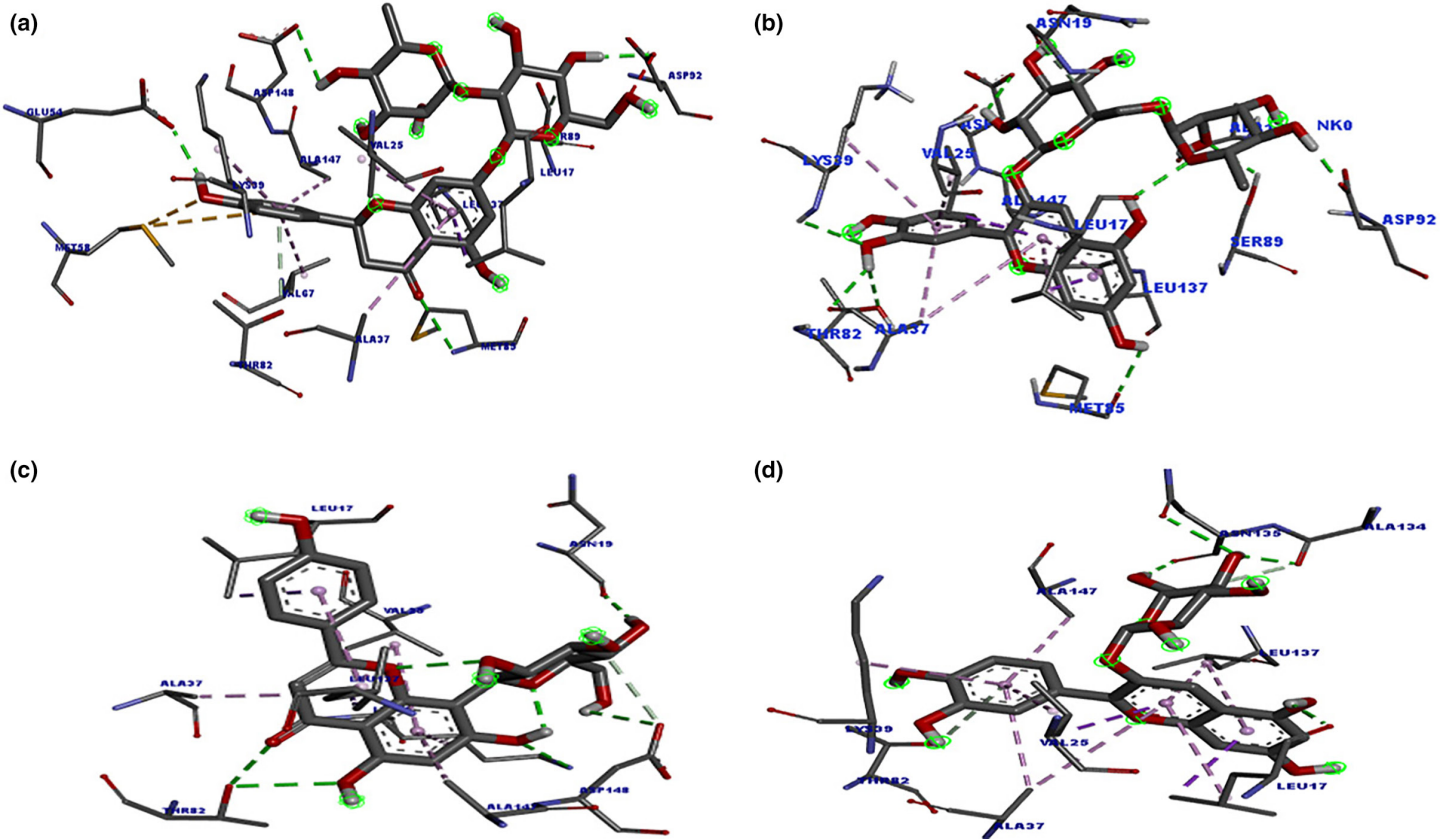
Binding pose predictions for the four ligands with the lowest ∆G values superimposed on the crystallographic structure of 2DQ7 target. (a) Naringin, (b) Keracyanin chloride, (c) Vitexin, (d) Cyanidin‐3‐*O*‐glucoside. Key hydrogen bonds are shown by green lines, and the protein molecular surface is colored by atom type.

Cytotoxicity analysis revealed that *T. coelesyriacus* natural compounds exhibited selective antitumor activity on pancreatic cancer cells. Pancreatic cancer‐specific marker proteins were investigated to elucidate the key players of such anticarcinogenic activity. The pancreatic cancer marker gene product FYN kinase was, therefore, targeted for in silico molecular docking analysis using *T. coelesyriacus* natural compounds. Docking analysis on FYN revealed that most of the native compounds of *T. coelesyriacus* showed a high binding affinity to FYN in the presence of low binding free energy. Among those compounds, naringin, vitexin, keracyanin chloride, luteolin, and cyanidin‐3‐*O*‐glucoside were identified with high binding affinity to FYN protein with low binding free energies, −10.1, −9.8, −9.7, −9.5, and −9.4 kcal/mol, respectively (Table 7). Among the analyzed compounds, *T. coelesyriacus* natural compound naringin was determined to have the highest binding affinity to FYN in the presence of the lowest binding free energy (−10.1 kcal/mol) (Figure 9). Furthermore, another ambitious inhibitor candidate for the FYN protein against pancreatic cancer cell lines was identified as keracyanin chloride with its high binding affinity, low binding free energy, and highest (8) H bond‐forming ability (Figure 10). Potential binding pose predictions of four natural compounds revealing high affinity for the 2DQ7 target in the presence of the lowest ∆G values were also investigated (Figure 11a–d). Among those, keracyanin chloride was identified as the hit compound with high binding affinity to the FYN kinase active site in the presence of a potential eight hydrogen bond interactions.

**TABLE 7:** Molecular docking data and interacting residue profiles of *T. coelesyriacus* native compounds when directed to FYN.

Compound id	Binding energy 2DQ7 (kcal/mol)	Interacting residues	H bonds
Chlorogenic acid	−8.1	**Chain X**: LEU17 ASN19 VAL25 ALA37 LYS39 GLU54 MET58 VAL67 ILE80 THR82 GLU83 TYR84 MET85 ASN86 LYS87 GLY88 SER89 ASN135 LEU137 ALA147 ASP148 PHE149	1
Cyanidin‐3‐*O*‐glucoside	−9.4	**Chain X**: LEU17 GLY18 ASN19 VAL25 ALA37 ILE38 LYS39 GLU54 MET58 VAL67 ILE80 THR82 TYR84 MET85 ASN86 LYS87 GLY88 SER89 ALA134 ASN135 ILE136 LEU137 ILE146 ALA147 ASP148	4
Epicatechin	−8.3	**Chain X**: LEU17 GLY18 VAL25 ALA37 LYS39 GLU54 MET58 VAL67 THR82 GLU83 TYR84 MET85 ASN86 GLY88 SER89 ALA134 ASN135 LEU137 ALA147 ASP148 PHE149	2
Ferulic acid	−6.2	**Chain X**: LEU17 VAL25 ALA37 LYS39 MET58 VAL67 ILE80 THR82 GLU83 TYR84 MET85 ASN86 GLY88 SER89 LEU137 ALA147	1
Fumaric acid	−4.3	**Chain X**: VAL25 ALA37 ILE38 LYS39 GLU54 MET58 VAL67 ILE80 THR82 GLU83 LEU137 ALA147 ASP148 PHE149	4
Gallic acid	−5.8	**Chain X**: PHE22 LYS39 MET46 SER50 PHE51 LEU52 GLU53 GLU54 ALA55 ASP148 PHE149 GLY150 LEU151 ARG153	N/A
Keracyanin chloride	−9.7	**Chain X**: ARG16 LEU17 GLY18 ASN19 VAL25 ALA37 ILE38 LYS39 GLU54 MET58 VAL67 ILE80 VAL81 THR82 TYR84 MET85 ASN86 LYS87 GLY88 SER89 LEU91 ASP92 ARG132 ALA134 ASN135 LEU137 ALA147 ASP148	8
Luteolin	−9.5	**Chain X**: LEU17 GLY18 VAL25 ALA37 LYS39 GLU54 MET58 VAL67 LEU69 ILE80 THR82 GLU83 TYR84 MET85 ASN86 GLY88 SER89 LEU137 ALA147 ASP148 PHE149	3
Naringin	−10.1	**Chain X**: LEU17 GLY18 ASN19 VAL25 ALA37 LYS39 GLU54 MET58 VAL67 ILE80 THR82 GLU83 TYR84 MET85 ASN86 GLY88 SER89 ASP92 ALA134 ASN135 ILE136 LEU137 ALA147 ASP148 PHE149	4
Quinic acid	−5.6	**Chain X**: LEU17 VAL25 ALA37 ILE38 LYS39 GLU54 MET58 VAL67 ILE80 THR82 GLU83 TYR84 MET85 LEU137 ALA147 ASP148 PHE149	4
Rosmarinic acid	−8.2	**Chain X**: LEU17 VAL25 ALA37 LYS39 GLU54 MET58 VAL67 ILE80 THR82 GLU83 TYR84 MET85 ASN86 LYS87 GLY88 SER89 ALA134 ASN135 ILE136 LEU137 ILE146 ALA147 ASP148 PHE149	4
Vitexin	−9.8	**Chain X**: LEU17 GLY18 ASN19 GLY20 GLY23 VAL25 ALA37 LYS39 GLU54 MET58 VAL67 THR82 GLU83 TYR84 MET85 GLY88 SER89 ALA134 ASN135 LEU137 ALA147 ASP148	5

## 4 DISCUSSION

**FIGURE 12:**
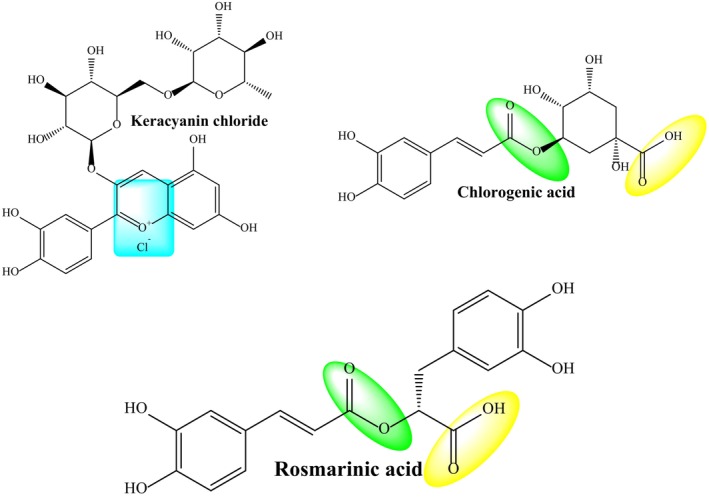
Polar regions of phenolic molecules in water and methanol extracts.

Leafy green vegetables are rich in bioactive phenolic compounds and have various biological functions, such as antioxidant, antimicrobial, and antitumor effects resulting from these phenolic compounds. These plants also contain large amounts of polyphenols, such as aromatic compounds, flavonoids, phenolic acids, and the most abundant phytochemicals in the human diet (Kim et al., 2013). As a result of our extraction study with *T. coelesyriacus*, 14 phenolic compounds were found by LC‐HRMS analysis; chlorogenic acid (13.58 g/kg) was the most prevalent in methanol extract, and quinic acid (8.79 g/kg) was the most prevalent in water extract. The amount of chemicals in the water extract is lower despite more phenolic compounds flowing into the methanol extract. The increase in the number of carbons in the structure of the compounds explains this. The enhanced solubility of compounds like naringin, ferulic acid, and luteolin in methanol, which is somewhat more nonpolar than in water, and the fact that these chemicals are not present in the water extract are due to an increase in the number of nonpolar carbons in the structures. However, even if only slightly, the passage of compounds into water extract can be explained by molecules like chlorogenic acid and rosmarinic acid, whose structure is as large as the others. This is because these molecules have functional groups like ester and carboxylic acid in their structures, increasing water solubility by adding extra hydrogen bonds. In addition, despite its complex structure, the keracyanin chloride molecule was also in the form of salt, although it was in a minimal amount in the water extract (Figure 12).

In the literature, various results were obtained in antimicrobial studies with different species of the genus *Tragopogon* (*Tragopogon dubius* Scop., *Tragopogon oligolepis* Hartvig & Strid, *Tragopogon porrifolius* L., and *Tragopogon graminifolius* DC.), with varying MIC values. In the in vitro study of Moromete et al., inhibitory effects of the two extracts obtained from the root, stem, and leaves of *T. dubius* were analyzed against *S. aureus*, *E. coli*, and *Streptococcus mutans* (for antibacterial), and *C. albicans* (for antifungal). It was determined that these extracts did not have an inhibitory effect on *C. albicans* and *S. aureus*, and the plant root extract had an inhibitory effect against *E. coli* and *S. mutans* at high concentrations (25%, 50%). MIC values could not be determined in this study performed with the disk diffusion method (Moromete et al., 2016). In the study of Farzaei et al., the antibacterial properties of extracts and the essential oil of *T. graminifolius* aerial parts were examined using disk diffusion and two‐fold microwell dilution methods. In the analysis against *S. aureus*, *K. pneumoniae*, *Proteus vulgaris*, and *Shigella dysenteriae* strains, the highest inhibition was found against *S. dysenteriae* with a MIC value of 2.5 mg/mL (Farzaei, Rahimi, et al., 2014). In the antimicrobial activity study of Uysal et al., ethyl acetate, methanol, and water extracts of *T. dubius* leaf were used. In this study performed by microdilution method, MIC (between 0.06 and 0.40 mg/mL) and the minimum bactericidal concentrations (MBC) (between 0.124 and 0.45 mg/mL) were determined for bacterial microorganisms. For yeast strains, the MIC and the minimum fungicidal concentrations (MFC) values were found to be between 0.02 and 0.18 mg/mL and between 0.03 to 0.25 mg/mL, respectively (Uysal et al., 2019). In another in vitro study, the antimicrobial activity of different extracts of aerial parts of *T. oligolepis* was investigated using the disk diffusion assay. The best inhibitory activity was seen in the ethyl alcohol extract of the plant against all Gram‐negative bacteria with an inhibition zone diameter of 11–21 mm and multi‐antibiotic‐resistant *Staphylococcus*. While the chloroform extract of the plant showed weak inhibition, the ethyl acetate extract did not show any inhibitory effect (Ugur et al., 2010). In the study using the microwell dilution assay, *T. porrifolius* antibacterial activity against *S. aureus*, *E. coli*, *Proteus mirabilis*, and *Enterococcus faecalis* was examined, and *T. porrifolius* showed activity only against *E. coli*, and MIC values were not determined (Tabaraki et al., 2013). The MIC value of *T. porrifolius* methanol extract against *Enterococcus faecalis*, *E. coli*, *S. aureus*, *P. aeruginosa*, and *C. albicans* was found between 2.5 and more than 2.5 mg/mL (Eruygur et al., 2019). The results above show that most *Tragopogon* species may have antimicrobial properties in studies with extracts from the *Tragopogon* genus. However, the methods used (disk diffusion, etc.) and the lack of MIC values in some studies have reduced their scientific value. Apart from all these studies, antimicrobial and anticancer properties of extracts of *T. coelesyriacus* were studied in our study. In conclusion, MIC values of the methanol extract of *T. coelesyriacus* against *S. aureus*, *E. coli*, *E. aerogenes*, *P. aeruginosa*, *K. pneumonia*, *C. albicans*, *C. tropicalis*, *C. krusei*, and *C. parapsilosis* were determined as 0.83, 53.3, 53.3, 1.67, 1.67, 53.3, 106.6, 0.83, and 26.6 mg/mL, respectively. In the antibacterial study, the methanol extract of *T. coelesyriacus* had the highest inhibitory effect against *S. aureus*, followed by *P. aeruginosa*, *K. pneumonia*, *E. coli*, and *E. aerogenes*, respectively. The differences between the antimicrobial properties of plant extracts against Gram‐positive and Gram‐negative bacteria result from the different cell wall structures of these microorganisms (Kim et al., 2013). *S. aureus*, a Gram‐positive microorganism, has a single layer of peptidoglycan. Due to its single‐layer structure, it does not show sufficient buffering against the protonation impacts produced by phenolic substances. Thus, bacterial cell cytoplasm may become acidified, and bacterial energy metabolism may be impaired. Nevertheless, lipopolysaccharides (LPS) and small membrane components in the cell membrane of Gram‐negative bacteria act as an inhibitory barrier against hydrophobic compounds. Therefore, they are more resistant to the antimicrobial effects of phenolic compounds (Helander et al., 1998). In addition, antifungal activity was observed in all *Candida* species, while the highest inhibition was seen in *C. krusei*, with a MIC value of 0.83 mg/mL. In contrast, the water extract of *T. coelesyriacus* had no inhibitory effect on the bacterial and yeast strains. Changes in the antimicrobial effects of *T. coelesyriacus* water and methanol extracts may have resulted from the differences in the phenolic compounds in the extracts.

Plant extracts usually contain antimicrobial compounds, and phenolic compounds form the main component of these antimicrobial compounds (Lin et al., 2004). It has been observed that phenolic compounds found in many plant species protect against pathogenic microorganisms (Walsh, 2003). –OH groups in phenolic compounds might be responsible for plants' antimicrobial and antioxidant effects (Gelssman, 1963). Phenolic compounds gain antimicrobial properties by causing dysfunction in bacterial cell membranes. The –OH groups in phenolic compounds are highly reactive in aqueous environments. It reacts with several biomolecules and causes the deformation of these molecules, thus preventing bacterial growth and reproduction. Phenolic compounds also bind to cell walls and proteins, inactivating bacterial enzymes and intercalating with bacterial DNA (Brooks et al., 1998; Pelczar et al., 1988). In this study, based on the LC‐HRMS data, it was found that both the number and the amount of phenolic compounds in the methanolic extract of *T. coelesyriacus* were higher than in the water extract. Almost 14 times higher chlorogenic acid in methanol extract (13.5768 g/kg) compared to water extract (0.9763 g/kg) may have increased the antimicrobial activity of the plant. Chlorogenic acid causes an important decline in intracellular ATP (adenosine triphosphate) concentration, possibly by affecting cell signaling and energy metabolism. It exerts a bacteriostatic effect by causing an intracellular metabolic imbalance of the glycolysis and tricarboxylic acid (TCA) cycle (Wu et al., 2020). Chlorogenic acid increases the plasma and outer membrane permeability, causing loss of barrier function and consequently disrupting bacterial cell membranes (Lou et al., 2011). In our study, phenolic compounds such as keracyanin chloride, cyanidin‐3‐*O*‐glucoside, vitexin, and rosmarinic acid in methanol extract more than in water extract may have affected the antimicrobial mechanism. Another study found that cyanidin‐3‐*O*‐glucoside showed outstanding antibacterial activity by showing a bactericidal effect against *S. aureus*. Changes in intracellular PH, intracellular ATP concentration, cellular morphology of *S. aureus*, and decreased membrane integrity caused cell membrane dysfunction (Li et al., 2022). The highest antibacterial activity achieved against *S. aureus* in our results may be associated with cyanidin‐3‐*O*‐glucoside, found in notable amounts in the methanol extract. Rosmarinic acid is a powerful antimicrobial agent that inhibits the growth of many bacterial and yeast strains and can inhibit the biofilm formation of microorganisms. It exhibits antibiofilm properties by destroying established biofilms and moderately reducing exopolysaccharide production. Rosmarinic acid exerts an antifungal effect by reducing mitochondrial activity, altering membrane integrity, and mildly inhibiting protease production in fungal cells (Ivanov et al., 2022; Kernou et al., 2023). In addition, in our study, three phenolic compounds (naringin, ferulic acid, and luteolin), which are present in the methanol extract but not in the water extract, may have contributed to the antimicrobial activity of the methanol extract (Table 1). Studies have shown that naringin, ferulic acid, and luteolin have serious antimicrobial activities by penetrating bacterial cell walls, damaging cell membrane integrity, or preventing biofilm formation (Céliz et al., 2011; Pinheiro et al., 2022; Qian et al., 2020). Ferulic acid is generally found in plant cell walls and isolated from the various parts of plants (Antonopoulou et al., 2022). The antibacterial activity of Moroccan *Mentha longifolia* leaf extracts was attributed to ferulic acid and other phenolics in the plant content, with the MIC value ranging between 1.17 and 12.50 mg/mL (Tourabi et al., 2023). The absence of these three phenolic compounds in the water extract in our study may be associated with the lack of antimicrobial properties of the water extract. Although fumaric acid is found in similar amounts in methanol and water extract, its antimicrobial activity has been proven (Unver, 2024c). Considering that most phenolic compounds contained in *T. coelesyriacus* have bactericidal effects, it is thought that the effect of *T. coelesyriacus* against microorganisms can be in this direction. Antimicrobial compounds form the basis of synthetic or natural drugs that can be used to treat infectious diseases. This preliminary study shows that *T. coelesyriacus* methanol extracts can be used as a natural raw material, antibacterial and antifungal agent, and therapeutic agent that can be developed for treating infectious diseases.

Our current molecular docking studies have provided useful information about the antibacterial potentials of *T. coelesyriacus* leaf extracts that add to previous findings. In addition, molecular docking studies have illuminated for the first time the remarkable inhibitory effects of keracyanin chloride on *S. aureus* MurB. In addition, the naringin molecule showed high binding affinity to *S. aureus* MurB in the presence of low binding free energy. Although the antimicrobial activity potential of naringin has been reported elsewhere (Jaradat et al., 2018), we report here for the first time the inhibitory activity of naringin on the *S. aureus* MurB enzyme. Our current molecular docking studies have provided information supporting previous findings. In sequence, molecular docking studies on *S. aureus* MurB elucidated its remarkable inhibitory effects of keracyanin chloride for the first time. Similarly, the naringin molecule showed high binding affinity to *S. aureus* MurB in the presence of low binding free energy. Both keracyanin chloride and naringin were able to show strong binding affinity to the active site of the MurB enzyme with six H bonds. In addition, two close Pi‐cation attractions and two weak Pi‐alkyl and one Pi‐Pi T‐shaped van der Waals interaction between residue Arg242 and the cyclic rings of keracyanin chloride further contributed to the *S. aureus* MurB and keracyanin chloride interaction. Differently, beyond the presence of six H bonds, naringin‐*S. aureus* MurB interaction involved two different negative donor–donor interactions and one each Pi‐anion and Pi‐alkyl interaction. Vitexin has also been identified with promising inhibitory activity on the *S. aureus* cell wall biosynthesis‐related enzyme MurB in the presence of −8.2 kcal/mol binding free energy. Similar inhibitory activity of vitexin on the biofilm formation of *S. aureus* pathogens has also been reported recently (Das et al., 2022).

Methanol extract of *T. porrifolius* was found to be cytotoxic in MDA‐MB‐231 breast cancer cells as well as in Caco‐2 colon cancer cells. Cell death was increased in a dose‐dependent manner from 5 to 100 μg/mL for 24 h and 72 h (Tenkerian et al., 2015). This study did not cover experimental data on normal cells to compare with cancerous cells. In vitro, the cytotoxic effect of ethanol extracts from herbs or roots of *Tragopogon pratensis* L. was shown in leukemic cells, and this effect was found to be correlated with the phenolic content of the extracts. IC50 after 24‐h incubation value was determined as around 300 μg/mL (Wegiera et al., 2012). Similarly, *T. pratensis* extract up to 100 μg/mL was cytotoxic in HepG2 hepatic cancer cells, but the death of normal cells (HaCaT) was also induced with the extracts (Kucekova et al., 2013). The species with cytotoxic effects shown from *Tragopogon* taxa are *T. porrifolius* and *T. pratensis* only. However, more species (such as *T. graminifolius*, *T. dubius*, *T. oligolepis*, and *T. porrifolius*) were shown to have an antimicrobial effect (Abdalla & Zidorn, 2020). To the best of our knowledge, there is no study revealing the anticancer activity of *T. coelesyriacus*. The present study suggests that both extracts of *T. coelesyriacus* showed a significant cytotoxic effect on AR42J pancreatic cancer cells. However, HeLa cervical and MDA‐MB‐231 breast cancer cells were found to be more resistant to the cytotoxic effect of methanol and water extract, respectively. Normal epithelial cells were not affected as much as cancerous cells, and their viability was around 80% after both extracts. In the literature, extractions were performed by a range of chemicals, including methanol (Warashina et al., 1991), ethanol (Farzaei, Khanavi, et al., 2014), hexane (Ugur et al., 2010), chloroform (Hariri et al., 2013), and water (Uysal et al., 2019). The contents of each extraction method can vary, and this can determine the effect of the plant. In our study, we found that the water extract of *T. coelesyriacus* does not have three components, including naringin, ferulic acid, and luteolin, compared to its methanol extract composition; however, water extract has more amount of four components, including fumaric acid, quinic acid, gallic acid, and epicatechin compared to methanol extract (Table 1). This difference can be suggested not to be deterministic for cytotoxicity in AR42J pancreatic cancer cells as the viability percentages of these cells reduced after both extracts. Nonetheless, the divergence composition may play a specific role in inducing cytotoxicity in HeLa and MDA‐MB‐231 cells. For instance, naringin, which is abundant in grapefruit, has been shown to regulate cell cycle arrest (Stabrauskiene et al., 2022) and to have a cytotoxic effect in HeLa cells (Martínez‐Rodríguez et al., 2020). Epicatechin was found to have anticancer activities on different breast cancer cells, including MDA‐MB‐231 and MCF7 cells (Pereyra‐Vergara et al., 2020). Our results confirm the previous findings on the specific cytotoxic effect of some metabolites on different cancer cells; however, this is the first study concluding the anticancer effect of *T. coelesyriacus* extracts.

Cisplatin has been widely used for cancer therapy so that it has been preferred to be used in in vitro and in vivo studies (Dasari & Tchounwou, 2014). Twenty micromolar cisplatin was previously found to induce cell death up to 48‐h treatment in AR42J cells (Uguz et al., 2012) and 25 μg/mL of cisplatin for 72 h promotes almost 90% cell death in HeLa cells (Liu et al., 2008). It was reported that viability of MCF10A cells was also negatively affected by cisplatin and some herbal medicines were shown to reduce cytotoxic effect of cisplatin on MCF10A cells during in vitro therapy (Kang et al., 2009). In this study, we found that the extracts of *T*. coelesyriacus were cytotoxic in cancer cells but not cytotoxic in normal cells suggesting that it is safe for healthy cells.

In the present study, the antitumorigenic potential of *T. coelesyriacus* extracts was also examined on three epithelial cancerous cells (AR42J, pancreatic cancer; HeLa, cervical cancer, and MDA‐MB‐231 breast cancer). A high binding affinity of *T. coelesyriacus* natural compounds on human pancreatic cancer marker protein FYN kinase was detected. FYN plays a crucial role in multiple cellular processes, such as cellular adhesion, motility, proliferation, apoptosis, cytoskeletal remodeling, integrin‐mediated signaling, immune response, and axon guidance (Sen & Johnson, 2011). Upon activation, it phosphorylates proteins involved in focal adhesions, ultimately regulating cell adhesion and motility (Posadas et al., 2009). Overexpression of FYN has been linked to the development of numerous tumors and metastasis, particularly in pancreatic cell lines (Peng & Fu, 2023). In addition, FYN influences various cancer‐related signaling pathways, making it a promising therapeutic target for tumor treatment. Suppression of FYN activity, thus, holds the potential to improve prognosis and extend patient survival in various cancer types (Peng & Fu, 2023). Recently, it has been reported that the SRC family FYN kinase is overexpressed in pancreatic cancer cells as a pancreatic cancer‐specific marker protein (Sen & Johnson, 2011). Experimental findings validated the selective cytotoxic activity of *T. coelesyriacus* extracts on AR42J pancreatic cancer cells. The pancreatic cancer‐specific FYN kinase was, therefore, targeted through in silico molecular docking analysis using compounds identified from *T. coelesyriacus* extracts. Among the tested compounds, naringin, vitexin, keracyanin chloride, luteolin, and cyanidin‐3‐*O*‐glucoside were identified with high binding affinity to FYN protein with low binding free energies, −10.1, −9.8, −9.7, −9.5, and − 9.4 kcal/mol, respectively. Beyond its promising antibacterial potential, molecular docking data also denoted the remarkable inhibitory potential of the naringin molecule on FYN kinase protein in the presence of −10.1 kcal/mol binding free energy and the establishment of six hydrogen bonds. 2D analysis of the active site revealed the involvement of various hydrophobic interactions between naringin and FYN Kinase residues. The relevant hydrophobic interactions were defined as two sulfur‐X interactions with Met58, one unfavorable acceptor–acceptor bond with Asp92, one van der Waals force with Ser89, and six Pi‐alkyl interactions with Val25, Ala37, Lys39, Val67, Leu137, and Ala147, respectively. Although the anticarcinogenic activity of naringin has been previously reported on various cancers, including breast, lung, stomach, prostate, and colorectal cancers, its effects on pancreatic cancer have been partially reported here for the first time (Stabrauskiene et al., 2022). On the other hand, the detection of vitexin, keracyanin chloride, luteolin, and cyanidin‐3‐*O*‐glycoside compounds with high binding affinity to FYN kinase suggests that their antitumor activities coordinately contribute to the anticarcinogenic activity of naringin in AR42J pancreatic cancer cell lines. The best‐docked conformation of the keracyanin chloride and human FYN kinase revealed that their biochemical interactions involve the formation of eight H bonds toward strong binding affinity. The best‐selected pose of keracyanin chloride had a binding energy of −9.7 kcal/mol. The 2D analysis of the active site further disclosed the availability of numerous hydrophobic interactions, including Pi‐sigma, Pi‐alkyl, and van der Waals forces, all of which eventually serve to the strong biochemical interactions between keracyanin chloride and human FYN kinase. In conclusion, compared to water extracts, *T. coelesyriacus* contains a high amount of certain compounds, including keracyanin chloride (18 fold), rosmarinic acid (15.5 fold), chlorogenic acid (10.2 fold), cyanidin‐3‐*O*‐glucoside (2.9 fold), and vitexin (1.5 fold). Although the docking studies pointed out that naringin stands as a promising anticarcinogenic compound within *T. coelesyriacus* methanolic extracts, its current quantity was quite low. Molecular docking studies thus further elucidated that the pronounced antimicrobial and anticarcinogenic activity of *T. coelesyriacus* methanolic extracts might be due to the high amount of those natural compounds, in particular, due to the high bioavailability of both keracyanin chloride and rosmarinic acid.

## 5 CONCLUSION

The water and methanol extracts of *T. coelesyriacus* were evaluated for their antibacterial, antifungal, and anticancer properties. The results indicated that the methanol extract of *T. coelesyriacus* has considerable antifungal and antibacterial activity. The bactericidal effects of most of the phenolic compounds contained in *T. coelesyriacus* extracts on microbial cells have been reported in the literature. Phenolic compounds, which are more intense in methanol extract, are thought to affect the antimicrobial activity test result. Furthermore, we can associate the different distributions of phenolic compounds in water and methanol extracts with the variability in the anticancer activity results. Both extracts from *T. coelesyriacus* showed anticancer activity, especially in pancreatic cancer cells, whereas normal cells were more resistant to cell death after treatment with extracts. In silico molecular docking studies support experimental data by elucidating inhibition of an antimicrobial protein (MurB involved in cell wall biosynthesis in *S. aureus*) and a cancer‐related protein (FYN) specifically increased in pancreatic cancer. Over the last century, the overuse of antibiotics, the resulting antimicrobial resistance, and the severe side effects of chemotherapeutic synthetics and antibiotics have led people to natural compounds, powerful alternatives. Extracts obtained from the edible plant *T. coelesyriacus* have the potential to be used as natural antimicrobial and anticancer therapeutics concerning the phenolic compounds it contains.

## AUTHOR CONTRIBUTIONS

**Tuba Unver:** Conceptualization (equal); data curation (equal); formal analysis (equal); investigation (equal); methodology (equal); project administration (lead); supervision (lead); validation (equal); writing – original draft (lead); writing – review and editing (equal). **Ugur Uzuner:** Data curation (equal); formal analysis (equal); investigation (equal); methodology (equal); software (lead); validation (equal); visualization (equal); writing – original draft (equal); writing – review and editing (equal). **Selcen Celik‐Uzuner:** Conceptualization (equal); data curation (equal); formal analysis (equal); investigation (equal); methodology (equal); project administration (supporting); validation (equal); writing – original draft (equal); writing – review and editing (equal). **Ismet Gurhan:** Investigation (equal); methodology (equal); validation (supporting). **Nur Sena Sivri:** Investigation (supporting); methodology (supporting). **Zeynep Ozdemir:** Data curation (equal); formal analysis (equal); methodology (supporting); validation (equal); writing – review and editing (supporting).

## FUNDING INFORMATION

This research did not receive any specific grant from funding agencies in the public, commercial, or not‐for‐profit sectors.

## Data Availability

The data that support the findings of this study are available on request from the corresponding author.
